# The M_w_5.4 Zagreb (Croatia) earthquake of March 22, 2020: impacts and response

**DOI:** 10.1007/s10518-021-01117-w

**Published:** 2021-05-08

**Authors:** Josip Atalić, Mario Uroš, Marta Šavor Novak, Marija Demšić, Miroslav Nastev

**Affiliations:** 1 Department of Engineering Mechanics, Faculty of Civil Engineering, University of Zagreb, Zagreb, Croatia; 2 Geological Survey of Canada, Quebec City, Canada

**Keywords:** Masonry buildings, Cultural heritage, Physical damage, Emergency response, Post-earthquake inspection, Social impacts

## Abstract

This paper highlights the principal features of the M_w_5.4 Zagreb earthquake. Located within the city limits at a depth of 10 km, the earthquake generated a peak ground acceleration of more than 0.2 g and a maximum spectral acceleration of about 0.6 g at 0.1 s in the historic downtown area. The situation was particularly challenging since the event occurred amid a partial Covid-19 lockdown at temperatures close to 0 °C, emphasizing the extensive and complex vulnerability of the local communities and individuals. 27 people were reported severely injured, one of which later died. The surprisingly high economic costs, needed to achieve a full reconstruction of damaged buildings and infrastructure in the affected area, are currently evaluated at more than 10B euros. Description of the organization of the emergency response in the first days and the observed damage to buildings is given with typical examples. The focus is on the performance of older masonry residential and cultural heritage buildings in the historic downtown, their inspection and evaluation of damage to structural and non-structural components. This information provides the basis for understanding of the negative impacts and clarifies the overall context identifying the enablers and barriers to the still ongoing recovery process. It also helps to increase the awareness of the seismic vulnerability of European cities with similar construction practices.

## Introduction

Strong shaking woke up the residents of the Croatian capital Zagreb and the surrounding areas at 6:24am local time on Sunday March 22, 2020. The magnitude M_w_5.4 earthquake (Dasović et al. 2020) was felt in northern Croatia and neighboring Slovenia and Bosnia and Hercegovina (EMSC url). The epicenter was located about 7 km north of the downtown area in the Markuševec and Čučerje neighborhoods, at a depth of approximately 10 km below the Medvednica Mountain (Seismological Survey url). The maximum perceived intensity at the epicenter was estimated VII on the EMS scale (Seismological Survey url), a strong earthquake with MMI intensity of VII (Markušić et al. 2020, USGS url). This was by far the strongest earthquake that hit Zagreb in the last 140 years, since the great M_L_6.2 Zagreb earthquake of 1880. The aftershock sequence was initiated immediately following the main event. At 7:01 am, the second earthquake of magnitude M_w_4.7 struck, and at 7:42 am there was another M_w_3.3 earthquake followed by a number of smaller tremors.

**Fig. 1:**
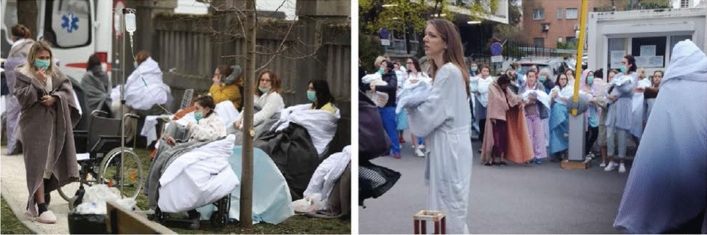
Evacuation of pregnant women and mothers with babies from the Clinic for Women's Diseases and Obstetrics in Petrova Str. (Photo: Government of the Republic of Croatia, 2020)

Despite the moderate intensity, the series of Zagreb earthquakes caused important social and economic impacts and damage to the built environment. Panic was widespread in the first hours and brought most of the population in the streets despite the Covid-19 lockdown. A total of 27 people were reported severely injured requiring hospitalization, one of which later died. The damage was extensive in the historic downtown (Lower Town, Upper Town and Kaptol). Walls and rooftops of older buildings sustained significant damage including the famous Zagreb cathedral dating back to the XIII century, which had been thoroughly reconstructed at the turn of the XX century. Streets were occasionally littered with falling debris from chimneys and façade walls damaging dozens of parked cars. Some districts faced partial power cuts and heating was disrupted due to the thermo-electric plant outage. Several hospital buildings were partially damaged, few of them beyond feasible repair, but all the others remained operational. The evacuation of patients from damaged buildings started urgently in the hospitals’ courts and then to the safer buildings nearby. The most shocking and iconic scenes from the Zagreb earthquake were seen in front of the old building of the Clinic for Women's Diseases and Obstetrics in Petrova Str. (Fig. 1). All patients whose medical condition allowed were discharged (IFRC 2020).

A state of emergency was immediately declared by the Civil Protection Authorities and the rapid response mechanisms were activated. Medical items, blankets, meals and hot drinks were provided through several distribution points to residents who could not return to their homes (IRFC 2020). A number of residents moved out of the affected downtown area, unofficial estimates vary from 15,000 to 20,000 people, whereas temporary accommodation was provided for about 500 people in a student dormitory. Fire and communal services together with units of the Croatian army were called to establish order and start clearing the city center and the surrounding streets. In parallel, teams of structural experts were sent to inspect and assess damage to buildings according to the predefined priorities. Following the inspection, the buildings were color tagged at a visible place. Residents were asked to strictly respect the tags and not to risk entering buildings with restricted access. The situation was particularly challenging the first days since the disaster occurred amid a partial Covid-19 lockdown, at temperatures close to 0 °C and strong winds. The schools were officially closed since March 16th and social distancing and quarantine were already put in place to prevent spread of the virus.

The objective of this paper is to provide a broad overview of the emergency response and of the negative impacts of the earthquake. It builds upon the initial findings reported by Šavor Novak et al. (2020), relevant scientific information published since the earthquake as well as the archived briefings from the government representatives and various first responders organizations. Large amount of collected field information and numerous data were still under processing and not available at the moment of drafting this paper. First, a brief description of the socio-economic setting and the seismological aspects of the Zagreb region are given together with the description of the main earthquake. The emergency response and the social impacts are reviewed next. The paper then focuses on the post-earthquake inspections, building safety evaluation and typical damage observations conducted in the aftermath of the earthquake.

## Zagreb metropolitan area

**Fig. 2:**
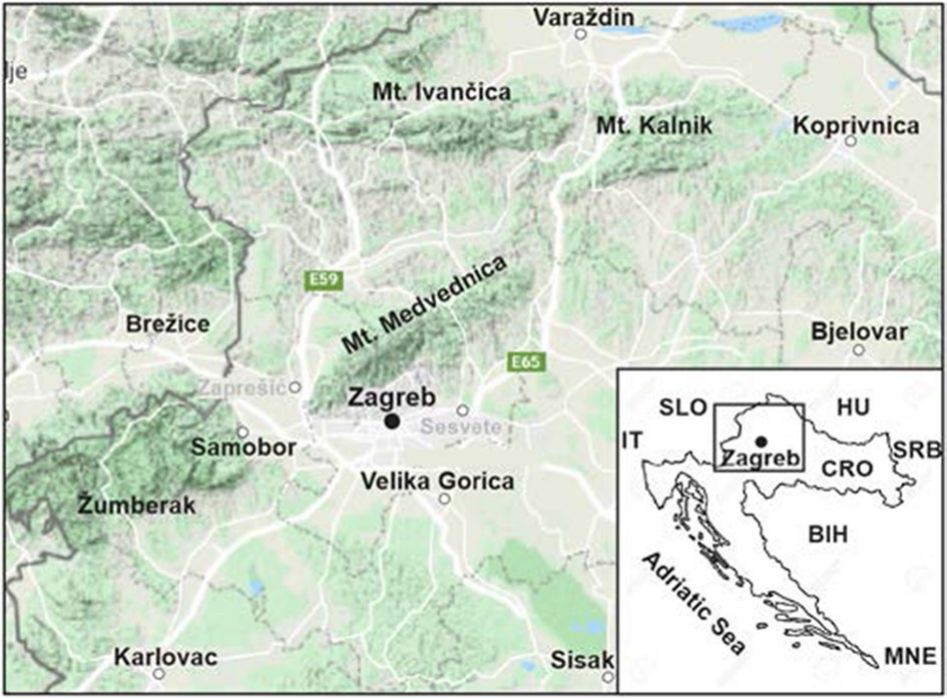
Location of the Zagreb region

Zagreb is the capital of the Republic of Croatia and a regional and cultural center of vital importance (Fig. 2). It houses critical administrative, educational, cultural and health institutions, commercial and industrial facilities and exceptional cultural heritage. Given the structure of the economy, industrial capacity and the city budget compared to other Croatian municipalities, Zagreb is considered the main economic and financial center. Census data indicate that about one third of the Croatian gross domestic product is concentrated in Zagreb (CBS 2020). The historic downtown is significant economically as a key tourist attraction in the country, where tourism contributes for about 20 percent of the overall GDP. On the other hand, due to the large number of state administration bodies, located mainly in the historic center, Zagreb is important for the political stability of the country. In addition, Zagreb is the national hub for road, rail and air traffic and a major intersection of European east–west and north–south routes.

According to the latest 2011 census, the City of Zagreb covers an area of 641.4km^2^ and has 790,017 inhabitants with 2019 projected estimate of 802,762 inhabitants, or close to 20% of the Croatian population (CBS 2019). There are 279,656 households occupying 334,888 private and collective dwellings. Zagreb is administratively divided into 17 city districts (CD) and 218 local committee areas (CA). The most densely populated CD is Donji Grad (the Lower Town) with about 40,000 inhabitants or 12,300 inhabitants/km^2^.

### Exposure

**Fig. 3:**
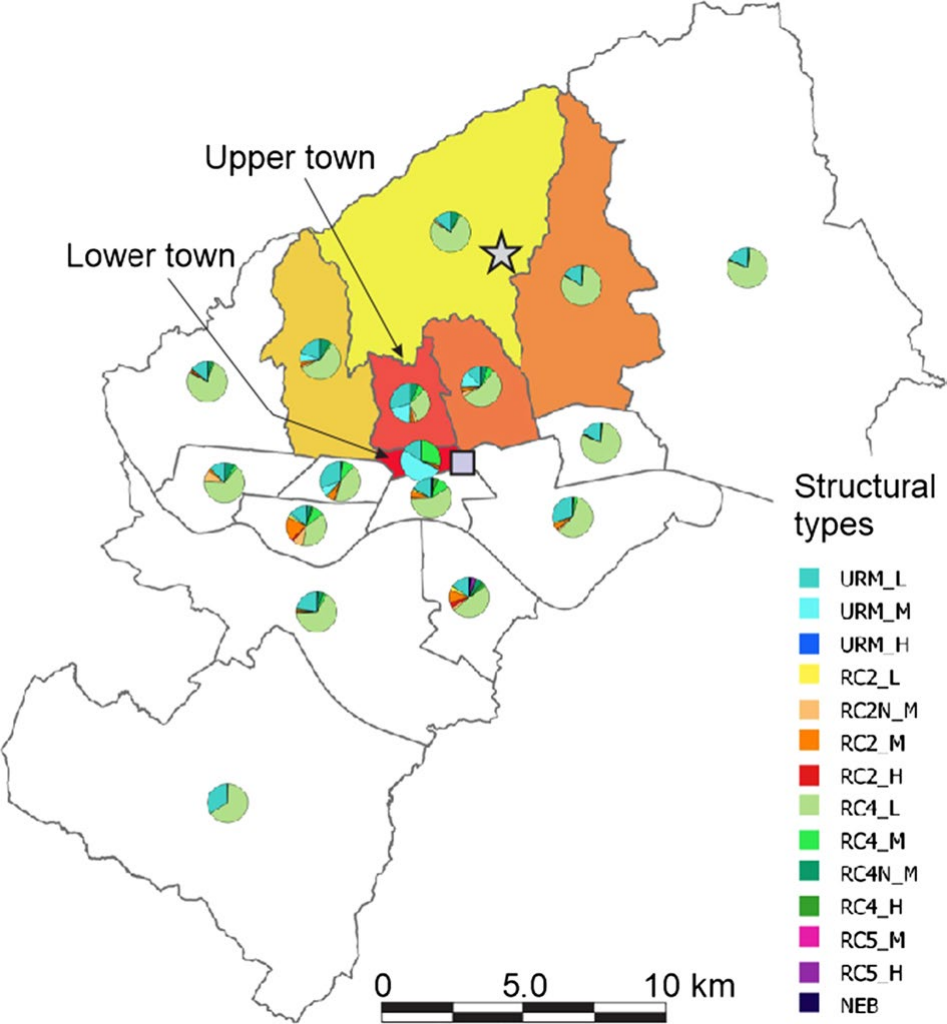
Zagreb map with distribution of buildings structural systems, aggregated at the city district level. The six most damaged districts are colored from lowest (white) to highest (red) number of damaged buildings. The epicenter (star symbol) and the seismological station of the Emergency Management Office (square symbol) are also indicated

The seismic risk assessment study conducted by Atalic et al. (2018) provides a detailed classification of the building types with respect to their structural system. In total, the existing 14 common types of buildings were defined including three average building heights (low L, medium M and high H): (i) unreinforced masonry buildings (URM) with wooden floor systems, vaults or reinforced concrete slabs in some floors; (ii) older buildings (approx. 1950–1980) with reinforced concrete walls (RC2); (iii) newer buildings with reinforced concrete walls (RC2N); (iv) older concrete frame buildings with infills and confined masonry (RC4); (v) newer concrete frame buildings with infills and confined masonry (RC4N); (vi) large panel reinforced concrete buildings, so called “cans” (RC5); and (vii) reinforced concrete high rise buildings (NEB). The distribution of buildings structural systems aggregated at CD level is shown in Fig. 3.

**Fig. 4:**
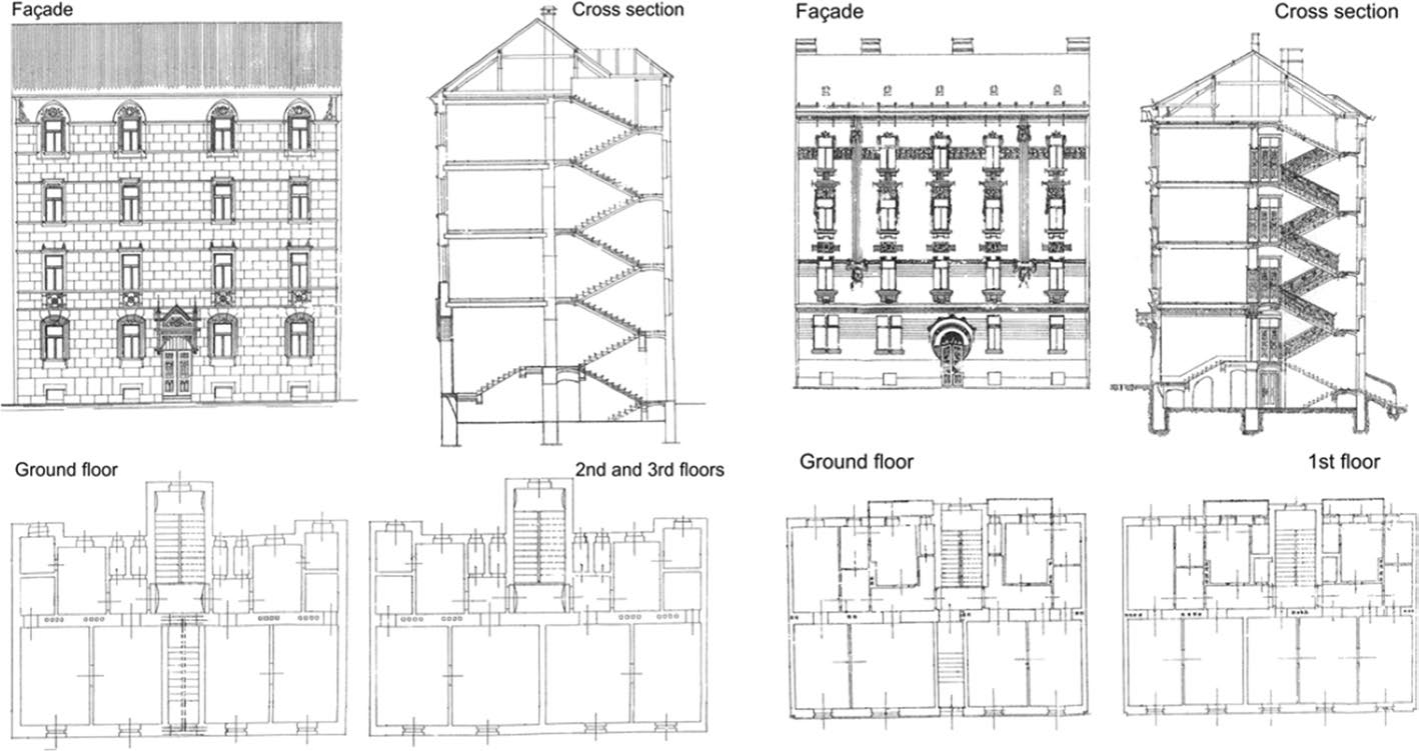
Typical examples of unreinforced masonry buildings from the beginning of the twentieth century in the historic center (after Crnogorac et al. 2020)

The focus herein is mainly on the older unreinforced masonry and heritage buildings, located in the historic downtown the most affected by the earthquake. In general, they were built during the reconstruction following the 1880 earthquake, consistent with the construction practices of the time (Fig. 4). According to the Office for Physical Planning of the City of Zagreb, the total gross built-up area in the historic downtown is approximately 5.2 km^2^, of which the buildings footprint is approximately 1.2 km^2^. The average number of storeys is 5 including the ground floor (Kiš-Bonačić et al. 2009). Most of the residential buildings were built in building blocks or as a row of buildings.

The fundamental period of vibration and the damping ratio are important structural parameters in the earthquake engineering. They help to estimate seismic forces and displacements of the structure commonly referred to as seismic demand. Measured in the elastic domain, the fundamental period of vibration depends on the mass and stiffness properties of the structure, and is also affected by structural irregularity, height, lateral dimensions of the building, etc. To better understand the dynamic properties of the URM buildings, a series of ambient vibration measurements were conducted. In Table 1 are given several typical examples of the fundamental period of vibration. Only the horizontal accelerations in the main directions were considered.

**Table 1:** Fundamental period of vibration of selected unreinforced masonry buildings in the historic downtown (after Atalić et al. 2020b)

Occupancy	Position	Number of stories	Footprint (m^2^)	Fundamental period of vibration (s)
Residential	Block of buildings	5	400	Tx = 0.39, Ty = 0.31
Educational	Single building	3	1250	Tx = 0.41, Ty = 0.31
Residential	Block of buildings	2	120	Tx = 0.10, Ty = 0.05
Residential	Block of buildings	3	170	Tx = 0.10, Ty = 0.08
Residential	Row of buildings	3	180	Tx = 0.10, Ty = 0.08
Residential and commercial	Single building	5	1100	Tx = 0.24, Ty = 0.16
Industrial	Single building	3	400	Tx = 0.25, Ty = 0.20
Medical care	Single building	5	1000	Tx = 0.54, Ty = 0.51
Residential and commercial	Single building	4	900	Tx = 0.31, Ty = 0.25

It can be observed in Table 1 that the fundamental period of vibration of the surveyed URM buildings is mainly in the range between 0.1 and 0.5 s (2 to 10 Hz). In Eurocode 8, the lateral earthquake loads are implicitly correlated to this parameter (Bisch et al. 2011).

In addition to the design characteristics, their age and often inadequate maintenance contributed to the poor performance of these buildings. Aggravating factors are surely the subsequent renovations, upgrades and conversions of the occupancy. The original up to 60 cm thick solid brick load-bearing walls, composed of two to three rows of moulded clay bricks, are often reduced in thickness or partially or even fully removed at the ground level to install street store windows. Steel lintels are frequently installed to span the new or extended openings. As well, in certain cases in the upper floors, partition and even interior bearing walls are completely disregarded as part of the structure and removed to gain space. Such interventions result in unsupported walls, which were initially continuous in the vertical direction, or in walls unsupported out-of-plane significantly weakening the structural system. These interventions are seldom documented and the current condition of the building differs significantly from the original documentation.

### Seismicity

**Fig. 5:**
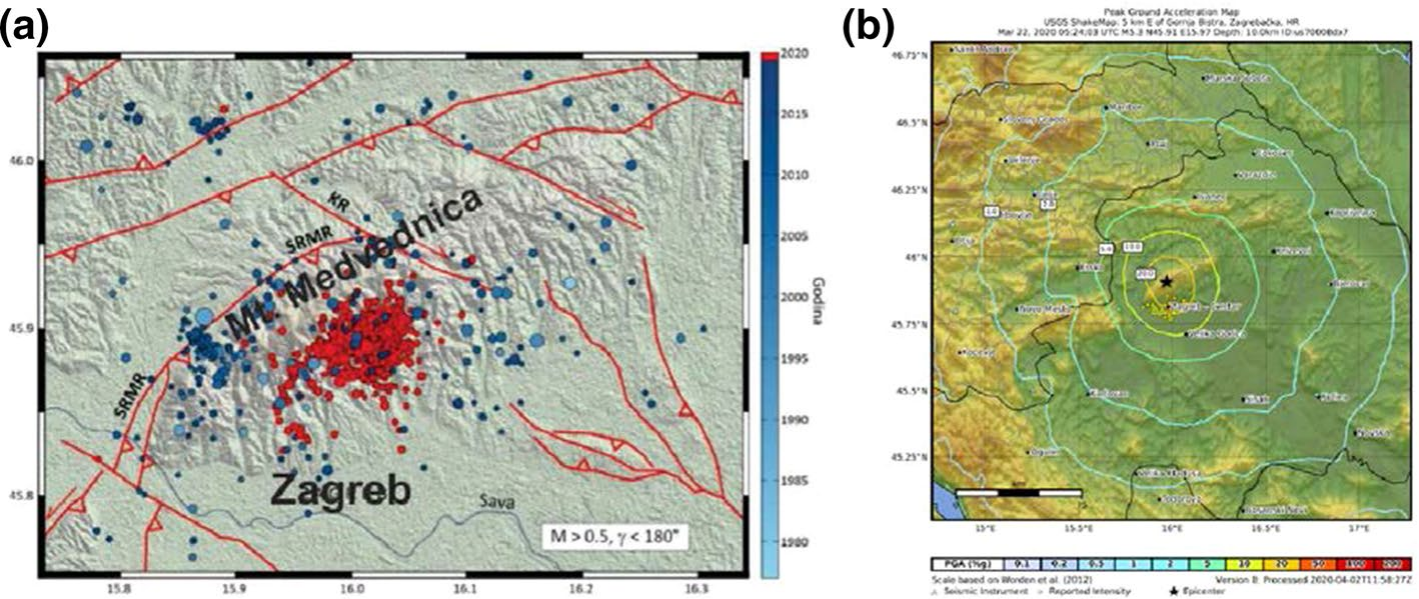
2020 Mw5.4 Zagreb earthquake: **a** major strike-slip and reverse faults in the Zagreb metro region (red lines), epicenters of M_L_ > 0.5 earthquakes since 1975 (blue circles), and epicenters of the series of 2020 earthquakes (red circles). Red arrows indicate direction of the relative slip, triangles indicate fault hanging walls, SRMR is the Northern marginal Medvednica fault, and KR is the Kasina fault ( modified from Herak et al. 2016); and **b** PGA shakemap (USGS url)

The Zagreb metropolitan area belongs to the wider seismotectonic region of the central-western Pannonian basin bordered by the mountain ranges of the Alps and the Dinarides to the west. The tectonic activity results from the interaction between the European and the Adriatic plates which generate non-uniform, in time and space, stress accumulations and releases within the individual seismic zones (Ustaszewski et al. 2010). Written records since the XVI century indicate about twenty earthquakes strong enough to cause significant impacts (Kuk et al. 2000). The seismic hazard in the local Zagreb metro area is controlled mainly by the nearby SW–NE-striking Žumberak–Medvednica–Kalnik fault zone (Markušić et al. 2020), which can also generate relatively strong earthquakes in the more distanced epicentral areas, e.g. Kalnik-Koprivnica, Brežice, Žumberak-Samobor (Herak et al. 2009). The seismotectonic aspects of the central part of the Žumberak–Medvednica–Kalnik fault zone, of interest for the present study, are described in details by Herak et al. (2016). In Fig. 5a are given the main faults in the vicinity of the Zagreb metro area. Among the most important active seismic sources are the reverse Northern Edge Medvednica Fault (SRMR), which extends along the northwestern edge of the Medvednica Mountain, and the approximately perpendicular Kasina strike-slip Fault (KR). Other reverse faults, such as the Sljeme fault, have also been identified below Medvednica Mountain (Tomljenović 2002).

The most important earthquake that struck the Zagreb metropolitan area is certainly the great 1880 M_L_6.2 earthquake with an epicenter in the Medvednica Mountain. It caused substantial destruction with practically all of the buildings damaged to a certain degree and about 13% collapsed or buildings damaged beyond reparation (Simović, 2000, Atalic et al. 2019). This resulted in a partial depopulation of the city, which took local authorities more than 25 years to reconstruct.

### The M_w_5.4 2020 earthquake

The epicentre of the main shock with magnitude M_w_5.4 is located about 7 km north of downtown Zagreb in the Markusevec and Cucerje neighborhoods, at a depth of 10 km below the Medvednica Mountain (Fig. 5). An aftershock sequence was initiated immediately following the main shock with more than 1000 tremors recorded within the next month. The strongest aftershock with magnitude M_w_4.7 struck approximately 40 min after the main shock and was followed by another M_w_3.3 aftershock at 7:42 am. Before processed earthquake records became available, Stanko (2020) and Markušić et al. (2020) estimated the average peak ground acceleration PGA at bedrock level in the downtown area at about 0.16–0.19 g. To account for potential amplification due to local site effects, the respective amplification factor for stiff soils was evaluated at 1.4–1.6 giving an average PGA of 0.22–0.3 g. The amplification factor for the peak spectral acceleration was estimated at 2.1. Similar values were predicted by the USGS shakemap (Fig. 5b), which implicitly considers site amplification based on the regional topographic slope as a proxy for seismic site-conditions.

**Fig. 6:**
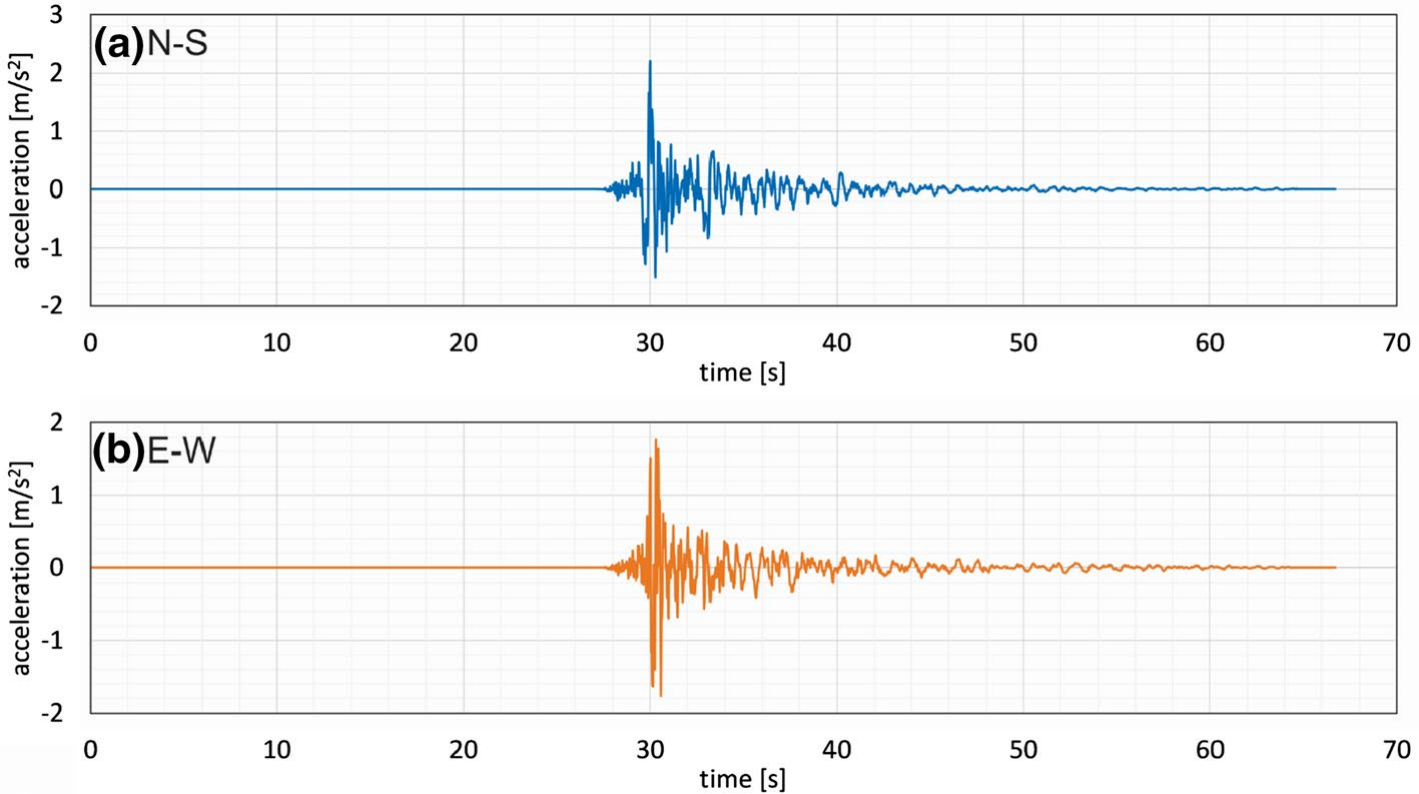
Recorded accelerograms at the Zagreb Emergency Management Office (EMO) station (Prevolnik et al. 2020) in horizontal directions: **a** N-S (blue) and **b** E-W (orange)

**Fig. 7:**
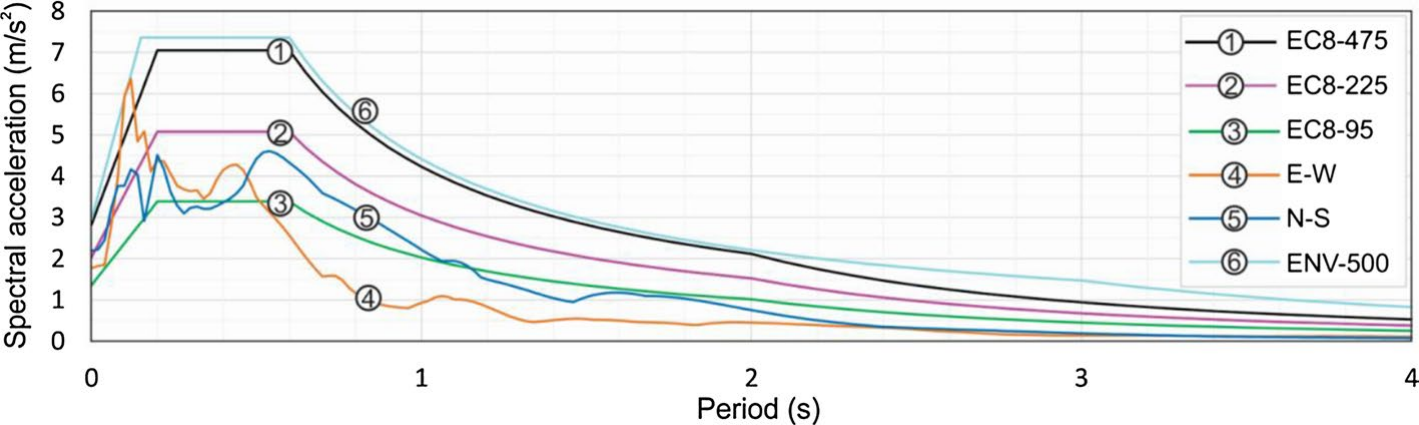
Spectral accelerations in horizontal N-S (blue) and E-W (orange) directions at the EMO station (location indicated in Fig. 3), respective Eurocode 8 (EC8) design response spectra for return periods of 95, 225 and 475 years, and the design response spectrum according to the former Croatian Seismic Code ENV-500 for return period of 500 years (modified from Šavor Novak et al. 2020)

There were two seismological stations and four accelerographs operating in a radius of 20 km around the epicenter, all maintained by the Croatian Seismological Survey (url). Accelerograms of the main event recorded at the Emergency Management Office station located about 8 km from the epicenter are shown in Fig. 6. The corresponding 5% damped pseudo-accelerations are given in Fig. 7 together with the Eurocode 8 design response spectra for three different return periods on soil type C for Zagreb (Herak et al. 2011). For comparative purposes, the design response spectrum according to the former Croatian Seismic Code (HRN-ENV 1998–1-1) for a return period of 500 years is also presented. It was derived converting intensity data from the seismic hazard map of Croatia, where part of Zagreb is situated in zone IX on the MCS scale. The respective base shear force was recommended for design of reinforced concrete structures, from 2005 to 2012, and of masonry structures, from 2007 to 2017.

Although generated by a moderate magnitude earthquake, these records indicate a horizontal PGA of about 0.2 g (Fig. 6). As expected, the energy content is concentrated in the higher frequency domain, 0.1–0.5 s, typical for a near epicentral region and stiff soils (Fig. 7). The E-W record, perpendicular to the dominant direction of wave propagation, from North to South, shows generally higher oscillations up to 0.4 s with a peak spectral acceleration of about 0.6 g at 0.1 s. This maximum spectral acceleration exceeds EC8 for 475 years and equals the old Croatian design response spectrum for return period of 500 years. The amplitude and frequency content of the earthquake motion (Fig. 7) provides useful information on the damage extent when combined with the fundamental period of vibration of the buildings (Table 1). The comparison of Table 1 and Fig. 7 shows agreement between the dominant period ranges and potential for resonance, which directly contributes to the increase of the destructive power of the earthquake.

## Emergency preparedness and response

### Organizational setup

To better understand the effects of the Zagreb earthquake and challenges faced in the immediate post-earthquake period, it is necessary to clarify existing enablers and barriers that have particular impacts on the resilience of the urbanized communities in Croatia. The main competences at the state level include overall legislation and execution, and security and defense. The municipalities assume responsibilities of local importance such as organization of settlement and housing, spatial and urban planning, utility services, primary health protection, protection and improvement of natural environment, fire safety and civil protection (Driver 2014). In this respect, the Zagreb Emergency Management Office EMO considers the earthquake preparedness on top of their priority list (EMO 2016).

Numerous activities related to post-earthquake emergency response and recovery situations, organization and education of intervention units, formation of urban search and rescue teams, field exercises, etc., contribute to increased resilience in case of natural disasters. The Croatian Platform for Disaster Risk Reduction within the Directorate of Civil Protection of the Ministry of Interior can be highlighted as a positive example (DRRP url). Its main objective is to foster continued cooperation between political, operational and scientific stakeholders to enable transfer and harmonization of knowledge, propose informed solutions, and adopt and ensure implementation of guidelines aiming at overall disaster risk reduction. Within this comprehensive system, the Zagreb Faculty of Civil Engineering was assigned as the lead scientific institution for activities related to seismic risk assessment. Experts from the faculty participated in training and civil protection exercises within various European projects (e.g., Matilda project url, Cascade’19 exercise url, etc.). As part of the Croatian technical-tactical support team, a few experts also participated in inspections of buildings safety following the 2019 Albania earthquake (Atalić et al. 2020a). The acquired expertise and knowledge proved essential during the March 2020 post-earthquake damage reconnaissance phase in Zagreb.

The public safety framework in Croatia is relatively well set, yet its capacity to cope in an emergency or disaster of higher proportions revealed insufficient. The Zagreb 2020 earthquake has helped identify certain gaps and needs for improvement in the response capacity and in the communication and synchronization between the state and municipal command chains. In the scope of their natural hazards risk assessment study, Atalić and Hak (2014) evaluated the impacts from a series of potential disasters in Croatia with the objective to prepare a common ground for comparison of respective risk management policies. The impacts from seismic scenarios exceeded by far potential losses from other disasters. The potentially disastrous consequences of strong earthquakes, beyond the criteria adopted by the European Commission, were further emphasized by Atalić et al. (2018). All these analyses recognized that Croatia lags behind countries with similar seismic settings, e.g., Italy, and that it should strengthen the organization of the emergency system. The authors recommended that the focus should be on activities that can be achieved relatively rapidly and without significant investments, e.g., implementation of operational policies and practices such as training exercises, development of methods for long-term and near-real time prediction of potential impacts and techniques for post-earthquake damage inspections. The ultimate goal would be to help reduce seismic risk by identification and application of feasible retrofit strategies for older buildings and strengthening of bridges, utility systems and other essential infrastructure components. The ongoing energy renovation and upgrading program for existing buildings has been identified as a potential path to follow (Stepinac et al. 2020). The need of a centralized comprehensive building and infrastructure database with linkages to the existing scattered databases and information was also pointed out as a major enabler to a more efficient emergency planning system. The Croatian Bureau of Statistics was invited to play important role in planning and acquisition of this strategic information.

### Emergency response

The March 2020 Zagreb earthquake occurred at the time of a sluggish economic growth in Croatia and amid the Covid-19 pandemic. The schools were officially closed since March 16th and social distancing measures were already put in place by the Croatian Civil Protection Headquarters. To contain the spread of the virus, people were urged via public media to avoid public gatherings and stay apart from each other. On March 19, three days before the earthquake, all public, religious and sport events were banned. The operation of services such as restaurants, shops and sport and recreation centers was suspended, with the exception of grocery stores and pharmacies. A temporary ban on border crossings was also introduced. The day before the earthquake, to avoid contacts, presence was limited in the streets and other outdoor public places where larger number of people gather or pass through at the same time, e.g., squares, bus stations, waterfronts, parks, etc., making situation even more unbearable and adding to the stress of the pandemic. A number of employees were advised to take annual leave and left Zagreb. It was estimated later that these measures, although unpopular at first sight, proved beneficial at saving lives.

**Fig. 8:**
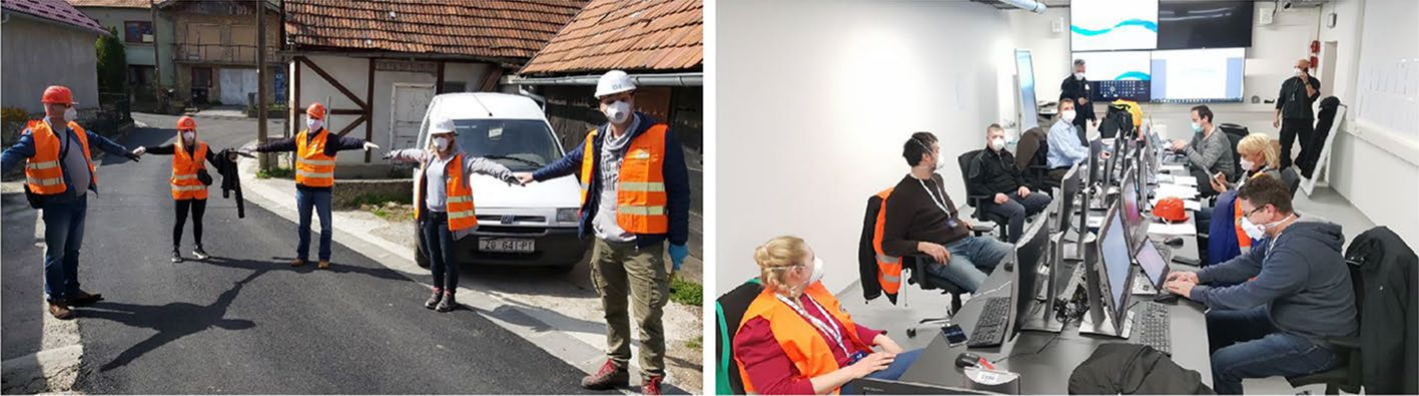
Headquarters for operational field management at the EMO and volunteers-experts in the field during the COVID-19 pandemic

Zagreb was much deserted on that early Sunday morning, a fortunate circumstance given the aftermath of the earthquake. Immediately after the mains hock, the Civil protection services were activated for emergency action. The members of the Zagreb EMO, the Directorate of Civil Protection of the Ministry of the Interior and of the Zagreb Faculty of Civil Engineering convened establishing the Crisis headquarters for operational management at the EMO (Fig. 8). Fire and communal services together with units of the Croatian army were called upon to maintain order and start clearing the city center and surrounding streets. Fortunately, the earthquake did not cause any major collapse of buildings or transportation facilities that would fully occupy the emergency services. The focus was therefore put on the assessment of damage and safety of affected buildings and infrastructure. Since there was no previously established inspection plan at city level, the technical experts self-organized using their experience and previous collaborations and under the guidance of experts from the Faculty of Civil Engineering.

As the scale of the destruction was unknown in the first hours all engineers who had undergone exercises and training for post-earthquake inspection of buildings were called upon by private calls. One of the first actions was to send them to lead the inspection of hospital buildings in the historic downtown, already identified as critical for post-earthquake recovery (Šavor Novak et al. 2019).

In parallel, at the EMO headquarters was initiated the fine adjustment of the initial safety and usability assessment methodology. Promptly, a general call was sent for mobilization of all engineers with expertise in the (1991–1995) post-war reconstruction or with knowledge related to traditional masonry structures. The Croatian Chamber of Civil Engineers was instrumental in providing the necessary support. On the first day alone, over 150 engineers voluntary responded to the call and started the inspection and assessment of building damage. The protective equipment (helmets, vests, etc.) required for safety during entry into damaged buildings together with masks, disposable gloves and disinfectants for Covid-19 were distributed to all first responders in the field. Programming of a mobile application (Collector for ArcGIS) for acquisition of field observations was initiated at the end of the first day; it was then tested the next day and put into operation a day later. The form was created according to the Italian (Baggio et al. 2007) and Greek (Anagnostopoulos et al. 2004) experience taking into account local building features and observed characteristic damage to gable walls, roofs and chimneys. All data was stored in a GIS based database for efficient information flow in both directions. For example, experts in the field could also retrieve data on the sick and self-isolated people due to Covid 19, important regarding the safety and limiting the epidemics spread.

In the first week, the number of volunteers rose to over 500 including volunteers-climbers for work at heights (e.g., roofs, chimneys). The emergency calls were monitored to ensure timely response and inspection in the order of arrival of requests. Requests for inspection were received also by e-mails and via the web page promptly established by the City for that purpose. Being rapidly overwhelmed, the frontline workers had to be reorganized, thereby responding on emergency basis and depending on the importance of the observed damage; it was already known that the hardest hit areas were the historic downtown and the epicentral neighborhoods of Markuševec and Čučerje. The damage assessment teams consisted of at least two structural engineers and/or architects with the number increasing with the size and occupancy of the building. Creating teams of experienced engineers, especially those who had undergone education and exercises, with younger fellows contributed to knowledge transfer and an increased inspection rate.

### Post-earthquake inspection

The work on post-earthquake damage inspection and assessment was coordinated by the Ministry of Construction and Physical Planning in cooperation with numerous partners from the government and the industry. The inspection of residential buildings was conducted visually and was more detailed in case of older masonry buildings and buildings that suffered apparent structural damage. Decisions on the short-term usability were made in discussion between the team members based on the current damage state and considering potential behavior of the structure in case a stronger shaking should have occurred during the still ongoing aftershock sequence. Decisions on usability of critical infrastructure (e.g., bridges) and of essential facilities (e.g., hospitals, schools) were made in agreement with the headquarters and people responsible for the institution. In both cases, the engineering experience and intuition were decisive for the evaluation of the safety and accessibility.

The first objective of the inspection was to identify and implement urgent measures to reduce to a maximum any potential risk of debris falling on neighboring buildings, sidewalks or driveways and other threats to human lives (e.g., collapsed chimneys, damaged façades and architectural finishing). The municipal representatives immediately warned people to beware of this potential threat and not to walk close to buildings. It was then important to restrain access to damaged buildings, to temporarily take care of people in need and to obtain as soon as possible a preliminary insight into the extent of the damage. Data collected in the field was practically immediately available to the fire and communal services, which then took appropriate measures of clearing and removal of debris. In addition, access to the database and insight to the buildings damage and usability reports was allowed to all relevant City services, e.g., firefighters, communal service, city offices, etc., and governmental departments depending on the level of authority. This allowed smooth exchange of information and transparency through daily briefings via public media.

The training given to a number of construction experts with focus on the typical damages observed in the field, proved to be crucial for the quality of inspections. First organized in-person at the EMO headquarters, given the pandemic circumstances, the training was later offered via internet together with accompanying handbook and webinars (CCEE url). The Croatian Centre for Earthquake Engineering CCEE website was intended to provide the necessary information to inspection teams in the field, to allow for a live monitoring of the status of inspected applications and was also used to prepare and educate citizens on the methods of building inspections. In addition, three WhatsApp communication groups proved effective for sharing essential information between field teams, including further training of younger engineers.

**Fig. 9:**
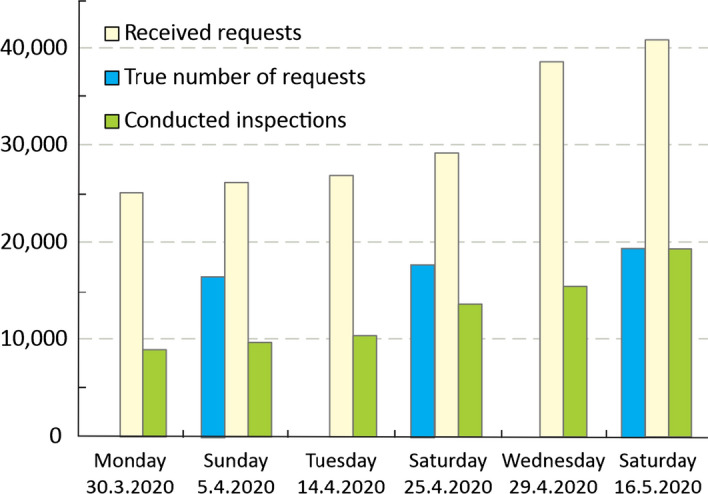
Number of received requests for inspection, the assessed number of “true” requests for inspection and conducted inspections (courtesy of the City Office for Strategic Planning and Development of Zagreb)

The site inspections encountered a number of unexpected difficulties. Some of the challenges faced by the field teams and crisis headquarters were frequent double-requests for inspection on the same address (Fig. 9), reaction of residents that were not familiar with the methods of inspections, residents that choose to stay home even when the building was red-tagged, conflicting and confusing information reported by the media, etc. Given the shortage of protective equipment on the market, it had to be procured in different ways, e.g., through numerous donations. Occasional shortages of protective equipment left frontline workers ill equipped rising concerns among the population. Due to closed borders and a ban on movement outside the place of residence, many engineers and specialists from other parts of Croatia and neighboring countries were unable to come to Zagreb to provide assistance. In addition, with the exception of external damage observations to roof structures and chimneys, modern reconnaissance technologies (drones, satellite images, etc.) could not be applied to the full extent, since most of the damage occurred inside the buildings (Stepinac et al. 2020).

The successful development and implementation of a feasible action plan for post-earthquake inspections was a challenge from the engineering point of view. Such plan did not exist before the earthquake and timely communication between the relevant institutions was particularly challenging in the first days of the post-earthquake activities. This also required a numerous adaptations of the GIS database to be able to respond to various needs (e.g., addition of necessary attributes, allowing access of different institutions to different attributes, etc.). The permanent communication between government and municipal representatives with citizens also revealed to be important for fine adjustments of the inspection activities.

## Spatial impacts

### Impacts on buildings

**Fig. 10:**
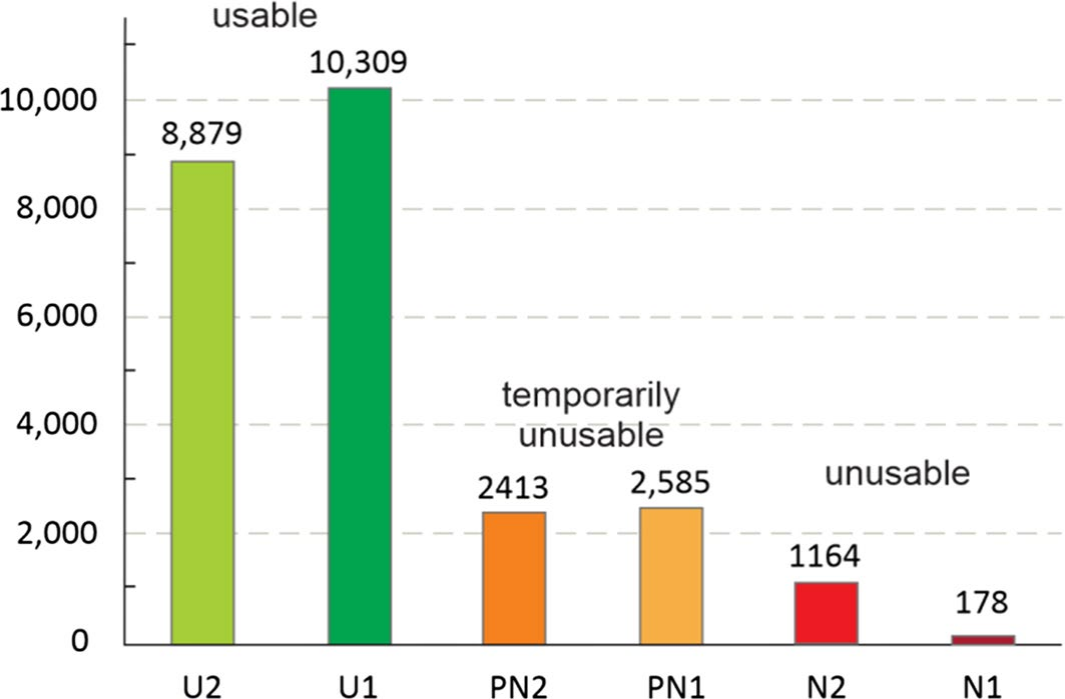
Inspection results and decisions on usability of inspected buildings as of June 30th 2020 when the preliminary assessments were officially finished

The post-earthquake field inspections of damage incurred to buildings were carried out until June 30th 2020, when the inspections were officially finished and the database was locked (GIS BUD 2020). Following the inspection, each of the buildings was marked with: (i) green (no structural damage observed; U1: entry is allowed without limitations, and U2: can be used with recommendation for short-term countermeasures), (ii) yellow (potential structural damage observed; PN1: temporarily unusable, require additional inspection, and PN2: can become usable after performing urgent interventions) or (iii) red (structural damage, no entry is allowed; N1: unusable due to external risks, and N2: unusable due to damage) color. Yellow- and red-tagged buildings require additional engineering review and the decision whether to repair or to demolish will be made based on the evaluation submitted to the city officials. The inspection data is still under processing and it is currently estimated that approximately 21,000 buildings were affected in a certain degree by the earthquake and many of them were inspected more than once. Detailed inspections results according to building usability are shown in Fig. 10.

**Fig. 11:**
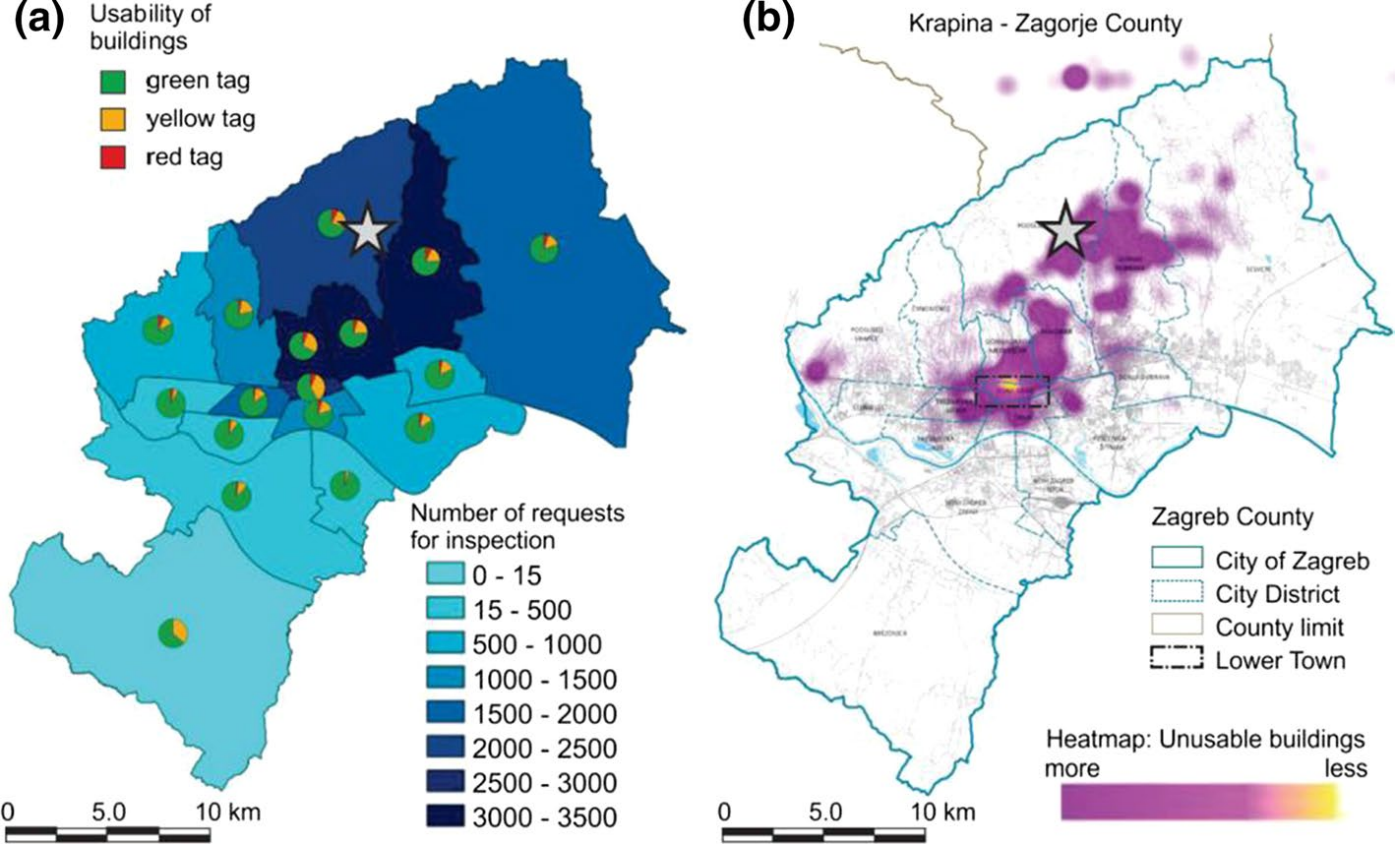
Impacts on buildings: **a** number of performed inspections (shades of blue color) and pie-charts of usability ratings across city districts (green-usable, yellow-temporarily usable and red-unusable; QGIS.org 2020); and **b** heat map of the unusable buildings. The epicenter is indicated with the star symbol (courtesy of the City Office for Strategic Planning and Development)

**Fig. 12:**
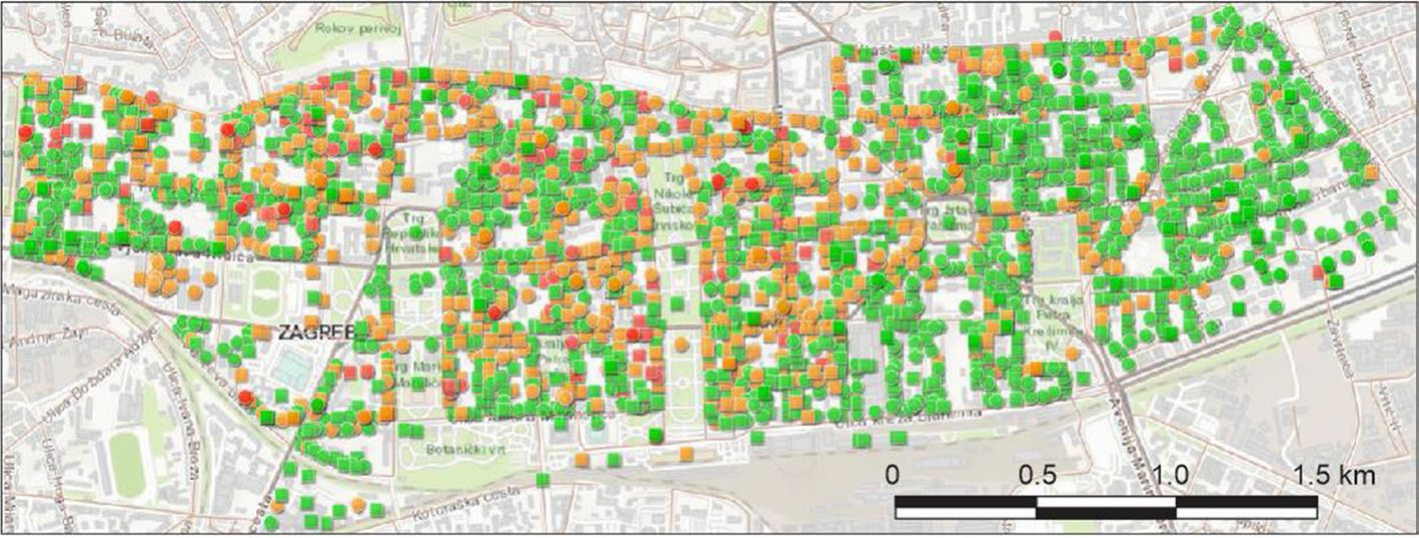
Building usability in the Lower Town (indicated with dashed line in Fig. 11): green-usable, yellow-temporarily unusable and red-unusable (GIS BUD 2020)

In total, more than 25,500 building inspections were performed or about 19.6% of the approximately 130,000 buildings within the city limits. Overall, about 75% of all inspected buildings were green tagged (U1 and U2), 20% temporarily unusable (PN1 and PN2) and 5% unusable (N1 and N2). The spatial distributions of building inspections at district level and building usability are given in Figs. 11 and 12.

Although of moderate intensity, the earthquake caused considerable damage to buildings. It may be observed that the highest concentration of inspected buildings and at the same time of the unusable buildings is located in the central city area and in districts close to the epicenter. The heatmap of unusable buildings provides information of the relative density of damaged buildings per unit area (Fig. 11b). Inspection results suggest that nearly all of the damage occurred in older masonry housing units and heritage buildings not designed to resist lateral dynamic loads. About one third of all buildings in Zagreb were built before 1964, when the first Seismic Construction Code was introduced. The vast majority of buildings built afterwards did not suffer any apparent impacts during the earthquake.

### Economic losses

A few different methods for evaluation of the renovation costs have been developed based on the World Bank methodology for loss assessment from natural disasters (GFDRR 2010). Results of the rapid damage inspections and information provided from different departments and city offices were considered. According to the preliminary analysis and cost projections for damaged buildings, which include family houses, apartment, residential and commercial buildings and public buildings, the direct costs for the renovation measures would be about 1.2B euros. The total economic cost related to complete renovation of the structure in the damaged buildings and to impacts to the already sluggish economy would attain more than 10B euros (Government of the Republic of Croatia 2020). These estimates were made relatively rapidly, about three months after the event, to activate the European Union Solidarity Fund. They indicate that most of the losses were sustained in the housing sector (64%), followed by the culture and cultural heritage sector (13%), education (10%), health (8%) and the business sector (5%).

It is difficult to obtain the exact monetary value of the economic losses when the reconstruction process is still ongoing, and even more difficult to make comparison with economic losses of other strong earthquakes that occurred in the region (Daniell et al. 2012). Beside the earthquake magnitude and distance, numerous factors contribute to the destructiveness of an earthquake: ground motion features in the epicentral region, structural types and density of the exposed buildings and infrastructure, respective vulnerabilities, reconstruction practices and their dynamics, socio-economic settings, etc. The aftershock series that strikes the already damaged buildings, the damage estimation models and reporting during field inspections are also very important. Here, economic losses of few damaging earthquakes in the region can be mentioned, e.g., 1963 M6.1 Skopje earthquake, North Macedonia (fatalities 1063; economic loss 5B euros), 1979 M6.9 Montenegro earthquake (136; 25B euros), 1997 ML5.9 Umbria-Marche earthquake (12; 5B euros), 2009 M6.3 l’Aquila earthquake (309; 15B euros), and 2012 M6.1 Emilia-Romagna earthquake (28; 13.2B euros) (Daniell et al. 2012; Dolce and Di Bucci 2017; Mircevska et al. 2019; Rossi et al. 2019).

Approximately six months following the earthquake, a planning law on reconstruction of the affected areas was adopted and accompanied by ordinances that define its implementation (Government of the Republic of Croatia 2020). Systematic interventions and specific measures have been recommended considering the assessment of the seismic performance and strengthening of the vulnerable cultural heritage buildings and essential facilities such as hospitals, schools and bridges. The performance and reconstruction of unreinforced masonry buildings in the historic downtown area is of particular focus. The measures aim at stabilizing out-of-plane failure of bearing and non-bearing walls, anchoring and tying of individual structural elements (e.g., walls and floor system) of the building with tension or shear anchors, replacing deteriorated mortar from joints with new mortar and removal of dangerous nonstructural elements (e.g., tall and heavy masonry chimneys). With a few exceptions, however, the systematic reconstruction of the affected buildings has not started yet. In the meantime, some of the residents had started taking measures on their own to repair and strengthen their properties.

## Typical damage observations

Residential buildings sustained most of the damage with almost two thirds of the total economic loss. Public buildings were also affected including a number of hospitals, schools, kindergartens, university buildings, student dormitories, the Parliament building, the buildings of the Supreme and County Courts, museums, theaters, sacral buildings, and many others.

### Impacts to health care facilities and education buildings

The safety of the health care facilities critical for post-earthquake response and recovery was even more important since the COVID-19 crisis was rapidly escalating. A total of 199 health facility buildings were located within the zone affected by the earthquake, out of which 42 health care centers, 115 hospitals and clinics, 20 health institutes and 22 pharmacies. Moderate to severe structural damage was reported for 39 buildings, including 8 buildings with heavy structural damage. Several important hospitals had to evacuate immediately patients and medical staff. The inspection of these buildings and the final judgement on the usability was very demanding for the experts because of the important consequences of their decisions, which sometimes had to include treatment interruption or withdrawal from life saving devices of non-ambulatory patients.

From the 42 clinical hospital buildings, 32 buildings reported with non-structural damages were tagged green with short-term countermeasures, 8 buildings suffered moderate structural and/or heavy non-structural damage and were assessed as temporarily unusable (yellow), whereas 2 buildings that sustained heavy structural damage were assessed as unusable (red). In addition to structural and non-structural damage, various levels of content damage (e.g., equipment) were reported in almost all hospital buildings.

Most of the Croatian health facility buildings date from the late 19th to early twentieth century. Around 80% of the buildings were built before the introduction of seismic considerations into the building code (before 1964), with only about 5% of buildings built after 2000. Additional 5% of the older hospital buildings have undergone structural interventions after 2000, though it may be questionable whether these interventions were beneficial for the seismic resistance. More than 60% of the hospital buildings are masonry structures built before 1960 without any reinforced concrete elements. In average, they have a basement and 3 floors and are either located in the cultural heritage protected area or are individually protected as cultural heritage. Hospital buildings with reinforced concrete load-bearing system are built after 1960s and are also mainly 3 stories high.

**Fig. 13:**
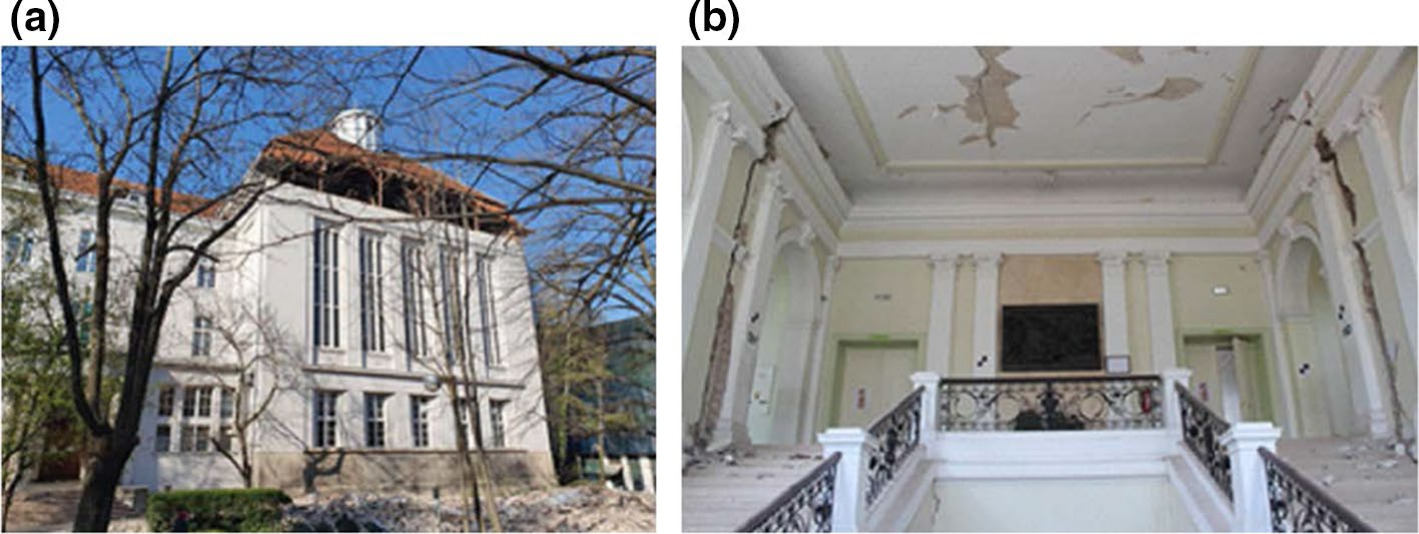
Damage to buildings of the Zagreb University: **a** School of Medicine and **b** Faculty of Law

As well, more than 500 education and research buildings were affected by the earthquake. Among them were about 200 schools, followed by preschool institutions and higher education, such as faculty buildings (28), academies (3), dormitories, etc. (Fig. 13). Fortunately, all these institutions were closed the day of the event. However, due to the damage extent, certain buildings could not be repaired and open before the next academic year and it was estimated that more than 6000 students would have to be relocated (Government of the Republic of Croatia, 2020).

### Impacts to heritage buildings

**Fig. 14:**
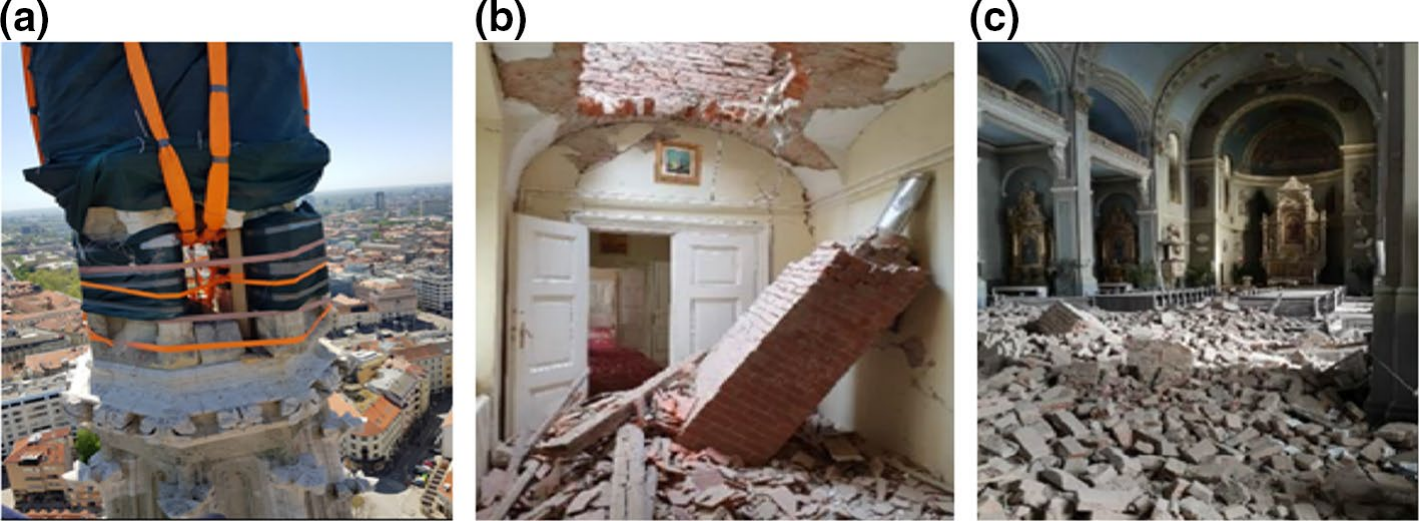
Damage to cultural heritage buildings: **a** removal of the bell tower spire, **b** interior of the Archbishop's Palace, and **c** Basilica of the Sacred Heart of Jesus (authors: Filip Foretić, Josip Atalić and Ivan Ćurić)

The earthquake also caused important impacts to the protected architectural and historical heritage buildings of inestimable value in the historic downtown. Of different occupancy categories, these old buildings represent a special cultural feature of the City of Zagreb. Almost 500 such were partially damaged with more than 10% red tagged. Among the cultural heritage buildings, the highest damage was sustained by centuries-old churches, 30 categorized as cultural heritage monuments. The first days, much attention attracted the threat of falling debris from these structures. The stone spire of the Zagreb Cathedral’s southern bell tower collapsed and caused damage to the roof of the cathedral. During the inspection, significant damage was observed to the spire of northern bell tower as well, which had to be urgently removed (Fig. 14a). In addition, the balustrade above the apse was destroyed, and the façade walls and vaults were significantly damaged. The Archbishop's Palace of the Cathedral sustained significant damage to vaults, roofs and collapsed chimneys (Fig. 14b). The Basilica of the Sacred Heart of Jesus had almost a third of its ceiling collapsed (Fig. 14c).

**Fig. 15:**
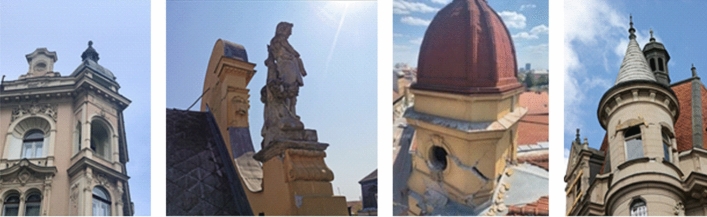
Damage to decorative elements (authors: Mario Todorić and CCEE 2020)

Architectural decorative elements of heritage buildings such as domes, portals, elaborately carved garlands and wreaths, sculptures, etc., suffered significant damage. When not adequately installed and anchored, these elements are usually unable to support lateral seismic forces. Due to their size and weight, they are made mainly of brick and/or concrete, they pose a serious threat to people’s lives. Examples of damage to these elements are given in Fig. 15.

There were numerous warnings from experts, even since the great Zagreb earthquake of 1880 (e.g., Mohorovicic), that decorative elements of heritage buildings pose significant threat to human lives, but were apparently neglected as were those more recent warnings with respect to the seismic performance of masonry buildings in general (Atalic et al. 2018, 2019). In the following text, the focus is on the performance of old masonry housing units in the historic downtown and causes and evaluation of the damage to their structural and non-structural elements.

### Performance of residential buildings

The old masonry housing units in the historic city centre were by far the most affected building type by the earthquake. Damage was most often caused by the lack of seismic conception, uneven stiffness distribution of vertical elements, flexible wooden floor systems which could not provide diaphragmatic behavior at the floor level, poor quality of the material (especially mortar), spandrels with low-quality of construction which affects the poor connection between the walls, and in particular the poor connection between mutually orthogonal load-bearing walls and contacts with the floor structure. In addition, frequent unprofessional reconstructions also affected their seismic performance. The most common observable external damage was the collapse of chimneys, gable attic walls and other cantilever parts at the top, and damage to the wooden roofs. In some cases, there was a significant separation of the gable wall along the height of the building and partial collapse of the façade wall. What could not be observed from the street view were damages to load-bearing and partition walls, and cracks at the contacts occurring mainly inside the buildings. Rarely, during the lateral movement, the wooden joists were completely pulled out from their sockets in the bearing walls and the floor structures collapsed.

**Fig. 16:**
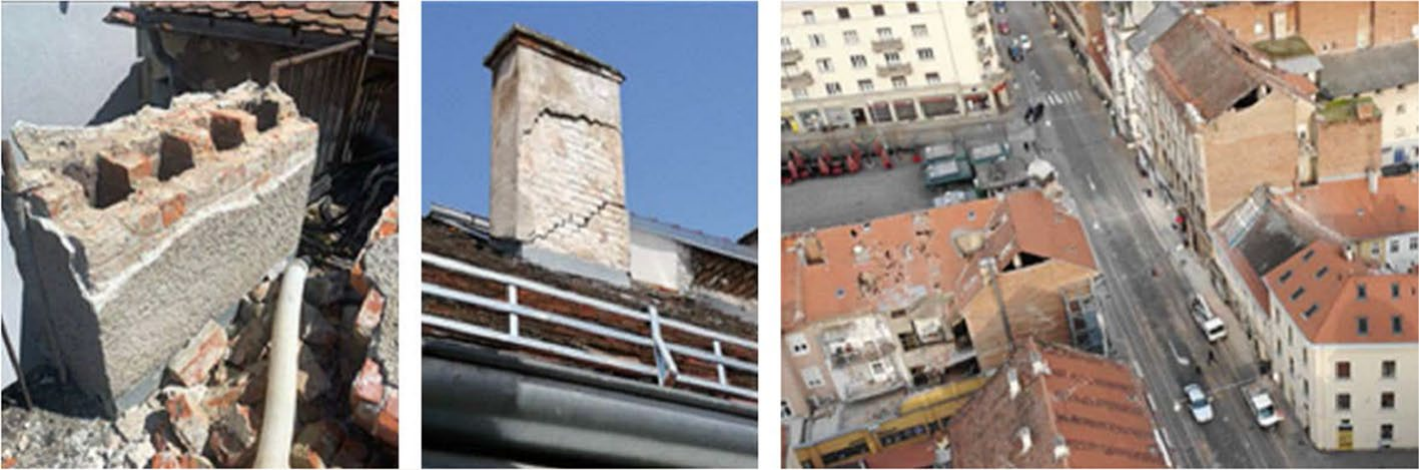
Typical damage to chimneys (authors: Mario Todorić and CCEE 2020)

*Damage and collapse of chimneys*: Much of the damage to roofs and attics were caused by the partial or complete collapse of chimneys (Fig. 16). Made of solid brick and mortar, they are not resistant to seismic forces. Chimneys are often free-standing starting from the attic floor, sometimes more than 5 m high, and only in rare cases they are fixed to the roof structure. Damage occurred mainly at the junction with the roof or with the attic floor structure (Crnogorac et al 2020). These types of chimneys make part of the Zagreb cultural heritage that was intentionally preserved, although it could easily be supposed, as it was the case, that their damage would frequently cause damage to roof structures and other building components and threaten occupants and people in the streets.

**Fig. 17:**
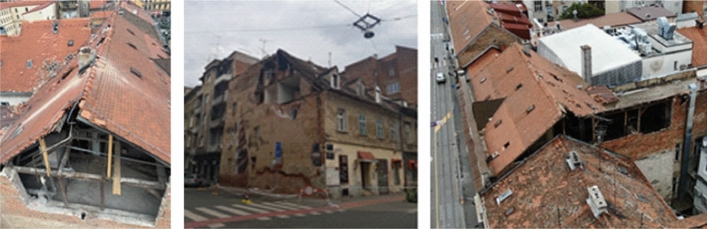
Typical damage to gable walls (authors: Mario Todorić and CCEE 2020)

*Damage and collapse of attic gable walls:* In addition to chimneys, the most common damage was the partial or complete collapse of the gable attic walls (Fig. 17). They are built as ordinary, mainly 15 cm thick masonry walls and usually not adequately supported with out-of-plane fixation to the roof structure. In certain cases, unsupported walls orthogonal to the attic gable walls were damaged, such as the longitudinal walls of the front and rear façades (Crnogorac et al. 2020). Additional weakness comes from the fact that the attic walls are not properly fixed to the floor structure; the wooden joists, spaced approximately 80 cm apart, are embedded in the longitudinal bearing walls without any structural connection to the attic walls.

**Fig. 18:**
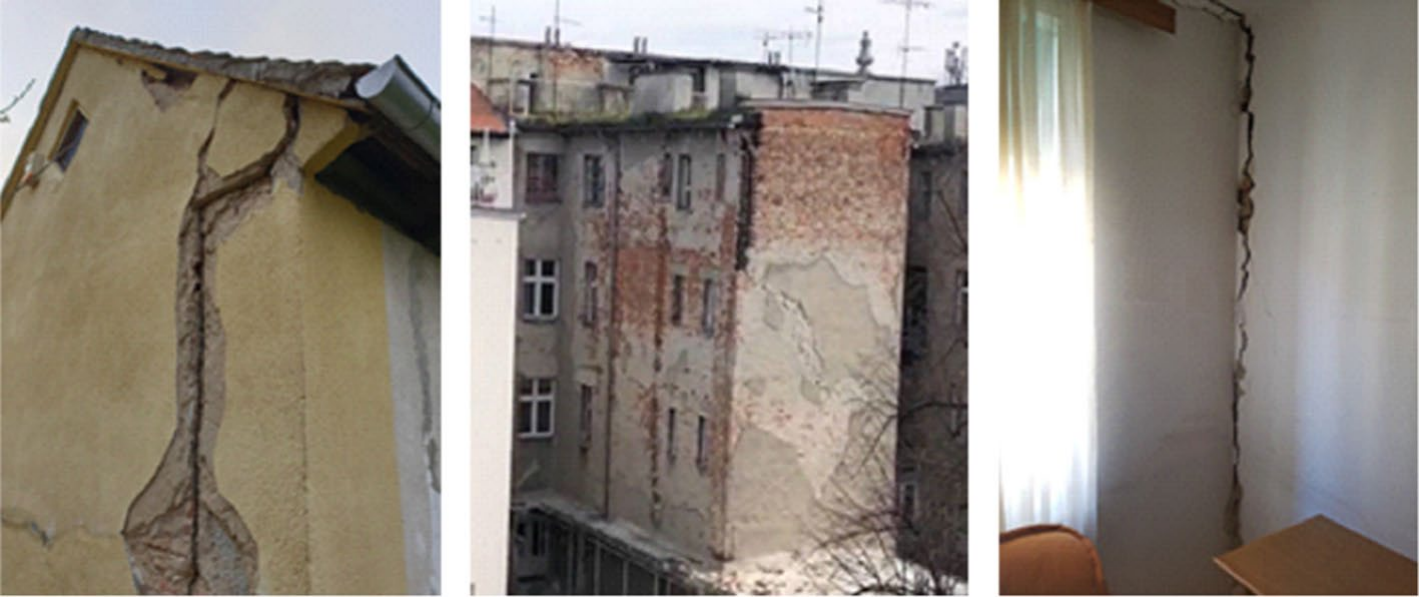
Separation of gable walls and characteristic cracks inside the building (authors: Mario Todorić, Mario Uroš and CCEE 2020)

*Separation of gable walls*: In severe cases, in addition to the local collapse of parts of the attic gable walls, a separation of gable walls developed along the entire height of the building or across a few storeys (Fig. 18). The reason was again the poor connection with the orthogonal bearing walls and with the floor structure resting on them. Therefore, the gable walls are not built to support much of the vertical load, which contributes to their poor resistance to lateral forces and the resulting differential out-of-plane movement. As a consequence, characteristic vertical cracks appeared in the orthogonal wall and horizontal cracks in the ceilings, mostly at the junction between the floor structure with and the gable wall. Increased separation can lead to overturning and collapse of the gable wall and may pose a danger to the whole structure and to the neighboring buildings as was often the case with tags N1 (unusable due to external risks).

**Fig. 19:**
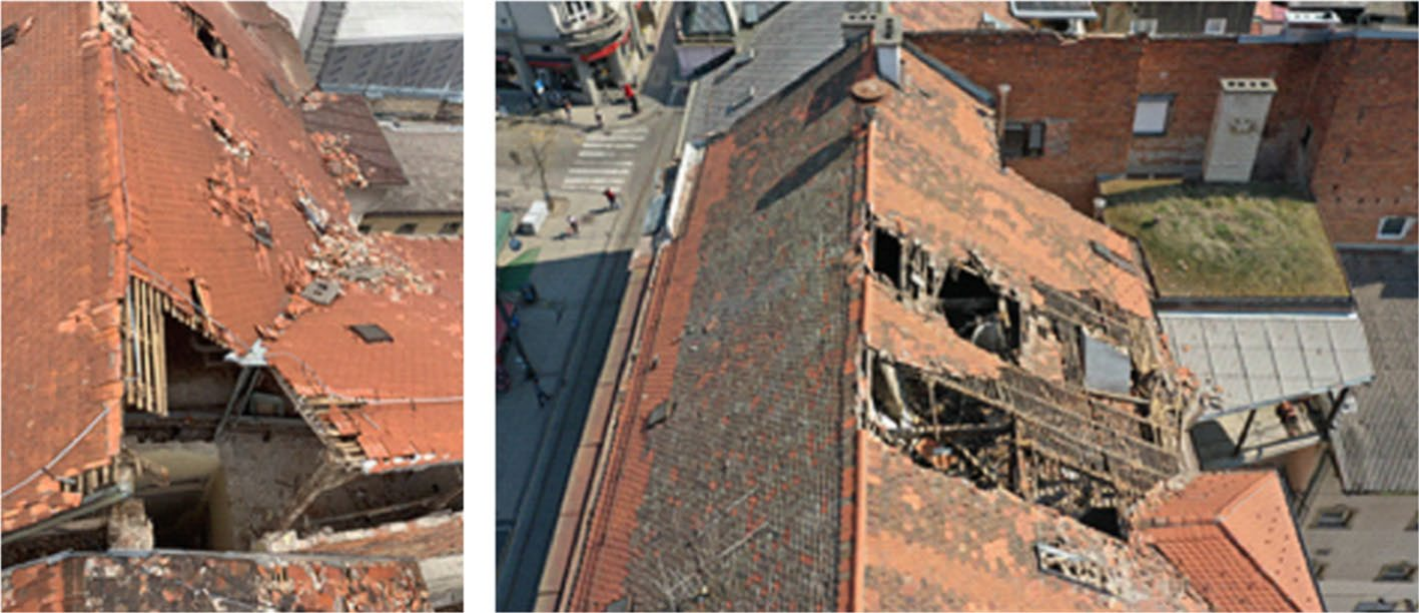
Examples of roof damage (authors: Mario Todorić and AIR-RMLD)

*Roof damage*: The most common cause of damage to the roof structure was incurred from collapse of chimneys, gable walls and other poorly supported elements above the roof surface. These represent mainly local types of damage (Fig. 19). The roof structures are usually of poor framing and diaphragm resistance and, due to lack of maintenance and wear with time, they provide unsatisfactory support to chimneys and attic gable walls. In certain cases, the entire roof structure sustained a permanent lateral displacement requiring a complete reconstruction.

**Fig. 20:**
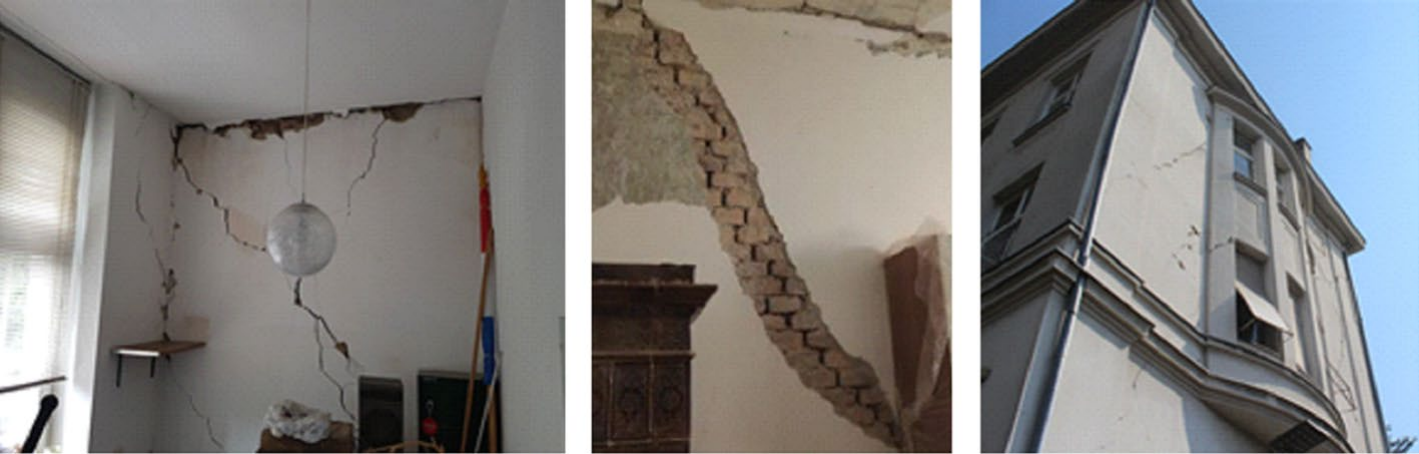
Typical diagonal cracks in load-bearing walls (photos: CCEE 2020)

*Damage to bearing walls*: Masonry load-bearing walls were most vulnerable to in-plane shear and out-of-plane bending culminating in an abrupt collapse at ultimate deformation. The deterioration of the structural material (brick and mortar) due to the long service life of these buildings (over 100 years), often poor maintenance and lack of façade finishing on some external walls, further endangers the stability of these structural elements. Typical diagonal cracks were commonly observed due to exceedance of the in-plane load-bearing capacity (shear strength) of the masonry walls. These were mostly stair-stepped joint shear failures (Fig. 20) due to the poor quality of the mortar, which has lost its mechanical properties. As this situation was widespread in damaged buildings in the city center, costs for repair and strengthening of the brick walls account for considerable part of the economic loss.

**Fig. 21:**
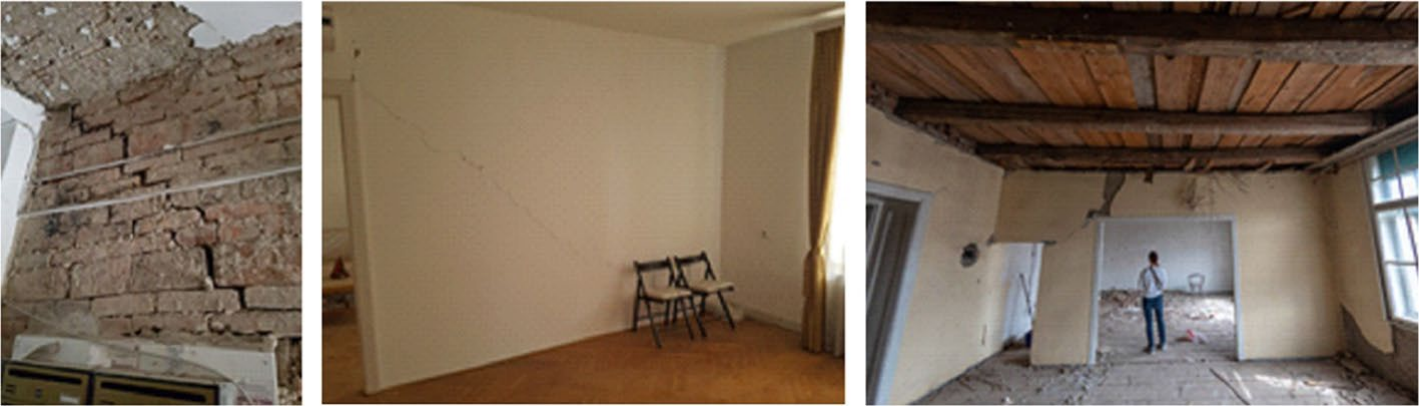
Typical cracks in partition walls (photos: CCEE 2020)

Non-bearing partition walls are mainly 15 cm or even only 7 cm thick. They are supported by wooden joists and are often not uniformly distributed in the vertical direction, what was expected after many adaptations and reconstructions. In case of stronger seismic loading, their contribution to the rigidity of the building is not substantial. As well, due to the low resistance but significant initial stiffness, they are unable to mirror the dynamic deformation of the entire structure and crack regularly. On the other hand, the partition walls constrain out-of-plane displacements of the orthogonal load-bearing walls to some amount, which is of importance to especially long walls. Significant number of damaged partition walls was observed in the direction perpendicular to the bearing walls; a few examples are presented in Fig. 21.

**Fig. 22:**
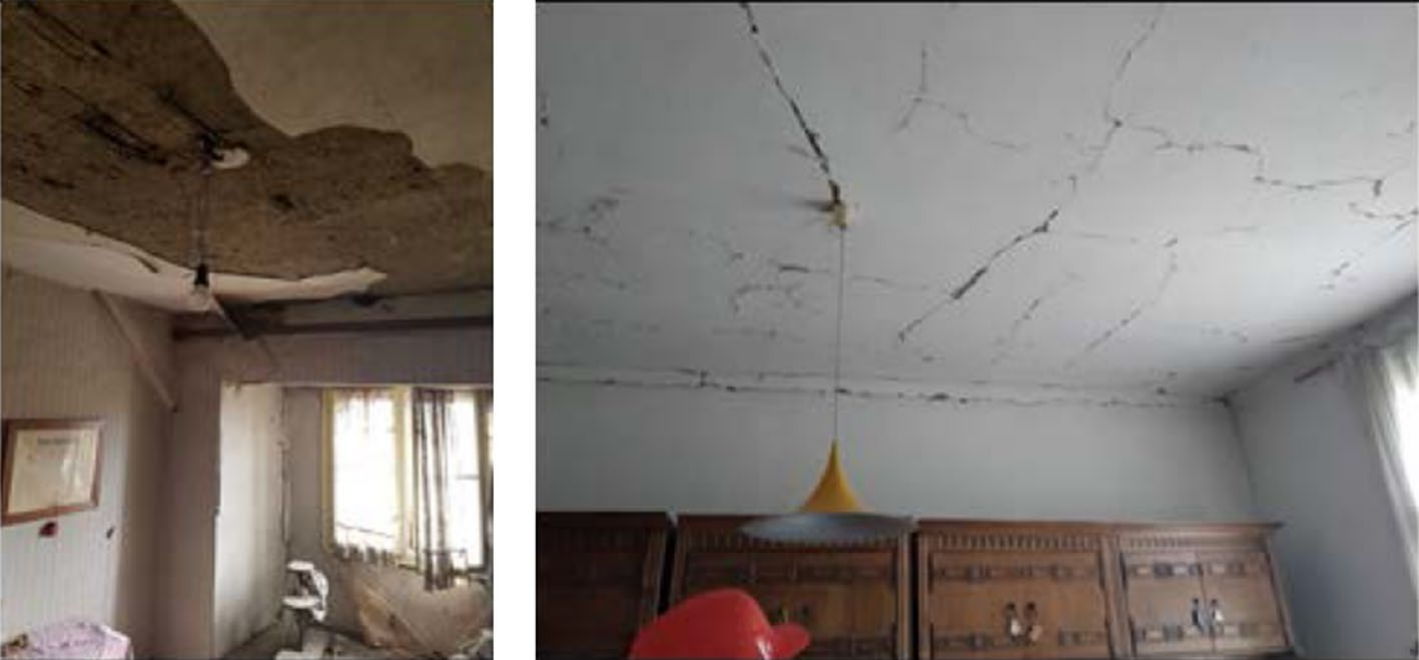
Typical cracks in the ceilings (photos: CCEE 2020)

*Cracks in the ceilings (floor structure)*: Cracks parallel to the direction of the wooden joists are most often due to the displacement caused by bending between the joists. Such cracks are within the plaster zone and represent seldom a structural problem as there are rarely tensile forces perpendicular to the joists. Due to the weight of the plaster, this type of damage may pose a threat to the occupants. Other cracks appear at the contact of the wall and the ceiling structure due to the relative displacements between these two elements. Basically, if there is no separation and tilting of the wall, it is again only a matter of cracking of the plaster itself as its tensile strength was exceeded locally (Fig. 22).

**Fig. 23:**
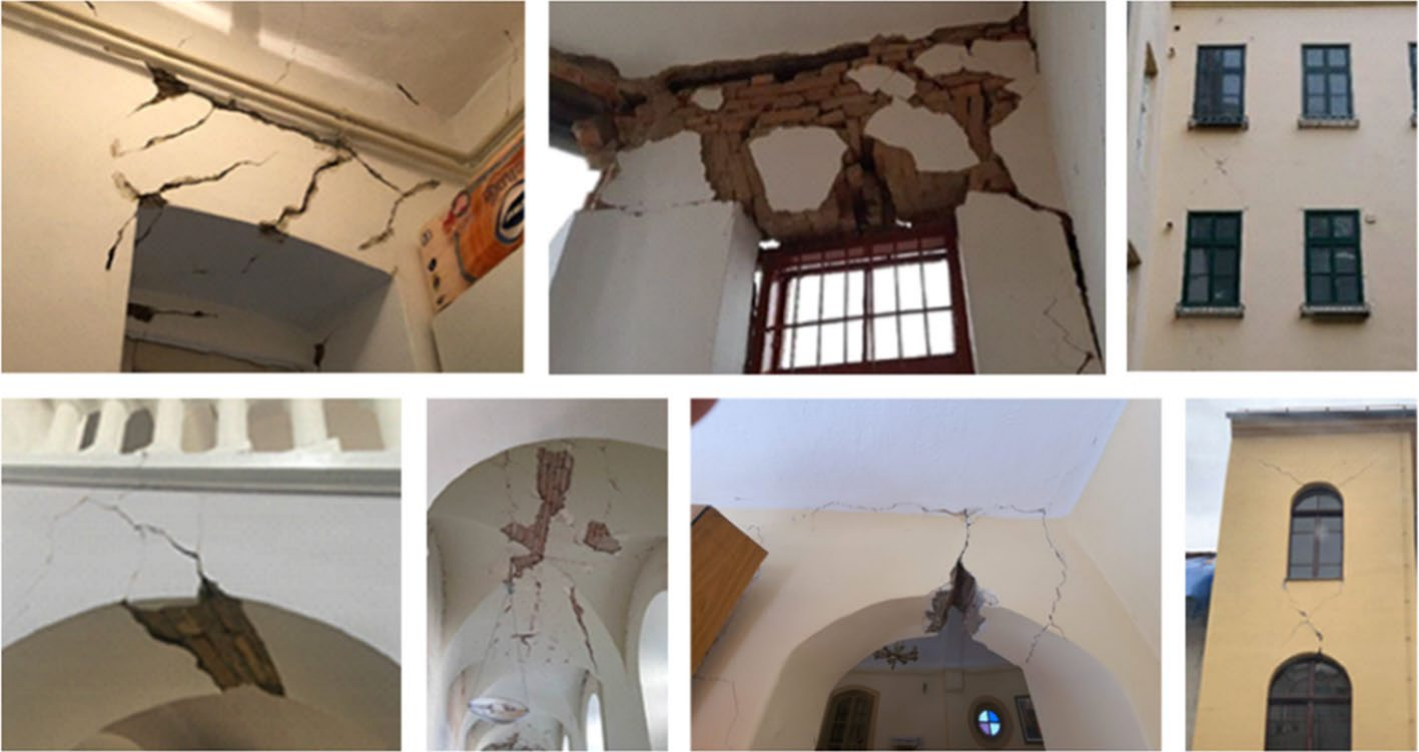
Cracking of the lintel and vault sections (photos: CCEE 2020)

*Damage to lintel and vault sections*: Another common form of observed damage was cracking of the lintel and vault sections (Fig. 23). In masonry buildings, openings are often bridged with horizontal wooden, brick or arched lintels. Diagonal cracks in the lintels were sometimes seen in the newer reinforced concrete buildings as well. When the cracks are relatively narrow and of limited extent, they do not necessarily mean any structural failure. However, larger cracks in some cases with material falling off indicate heavy damage to masonry lintels. This type of damage is dangerous and need to be secured against collapsing. Diagonal cracks in the lintel section are due to the exceedance of the shear strength. On the other hand, approximately vertical cracks in vaults are caused by stability disturbance and triggered by exceedance of the tensile strength.

**Fig. 24:**
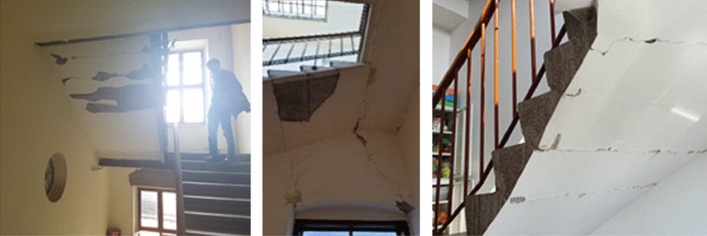
Damage to stairways (photos: CCEE 2020)

*Damage to stairways*: Stairways were often damaged in the masonry buildings, where relatively rigid core of thick masonry is found around the staircase (Fig. 24). The older cantilever type of staircase has been mostly replaced and stairs are now often supported by steel girders anchored in the landing zone. This type of damage refers mainly to structural damage in surrounding bearing/landing walls or is due to differential movement between adjacent floors and separation of structural elements. In order to render stairs usable, it was necessary first to clear and prevent additional debris falling in the stairwell.

**Fig. 25:**
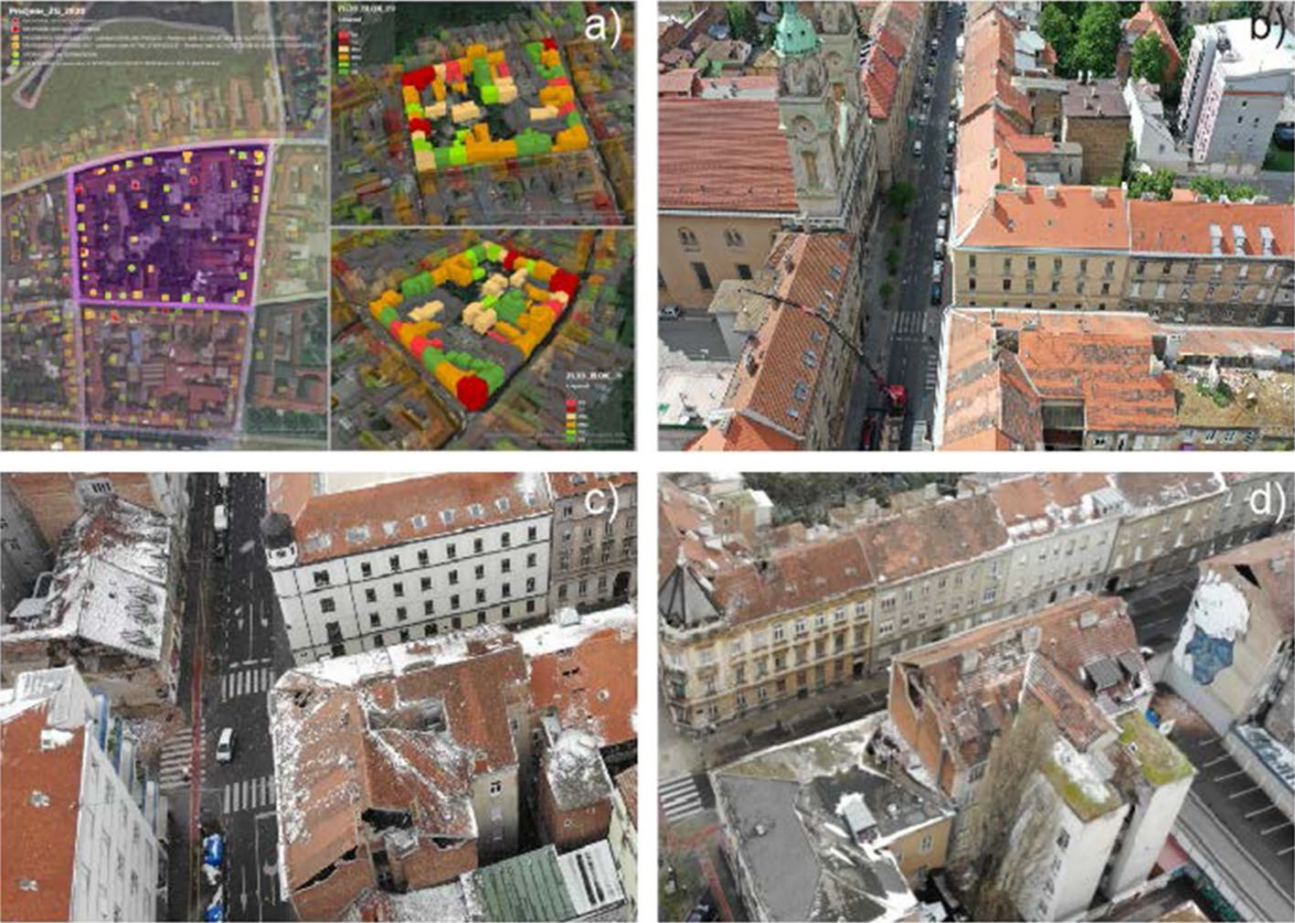
Examples of observed damage in building blocks: **a** distribution of damage to buildings in a building block (from green: no damage, to red: high damage), **b** buildings without a neighbor on one side, **c** corner buildings, and **d** buildings taller than the surrounding buildings

*Damage to buildings in blocks*: As mentioned earlier, the buildings in the historic downtown are built in relatively regular blocks of 5 or more buildings of varying dimensions, 100 × 50 m in average (Fig. 25). The sidewalls of these buildings are adjacent to each other and, although there are no common gable walls, they often lean against without any partition. Due to frequent protrusions for drains and/or used water piping, windows, or due to various interventions over time, the gable walls of adjacent buildings are often wedged or joined together. It can therefore be assumed that buildings that are not located at the corner of the block behave more or less as interconnected. The width of the building determines the spanning direction of the floor joists. In general, the floor slabs are supported by the longitudinal walls parallel to the street. This is the stronger load-bearing direction for lateral seismic forces, whereas in the perpendicular weaker direction the stairwell, separation and/or gable walls are unsuited to support significant loads.

The concept of ‘block of buildings’ appeared to be less vulnerable to earthquake loading contributing to fewer damage observations during inspections (Fig. 25). The structural characteristics at the contact with the neighboring buildings, however, remain uncertain and there is a complex interaction in the dynamic response. This further complicated the identification of the cause of damage. Buildings at the corners are generally built with load-bearing walls in both directions, a system which provided relatively high seismic resistance. Problems were noticed in buildings that exceed the height of the rest of the buildings, a common construction practice in Zagreb. There, the damage was concentrated to the prominent "free" floors. Buildings without a neighbor on one side were also susceptible to earthquake damage. Such was the case when the building row was interrupted due to demolition of dilapidated buildings thereby freeing-up space for car parking and access. Damage to these buildings was mainly observed on the free side walls, and occasionally in certain walls within the floor plan and walls facing the neighbor.

**Fig. 26:**
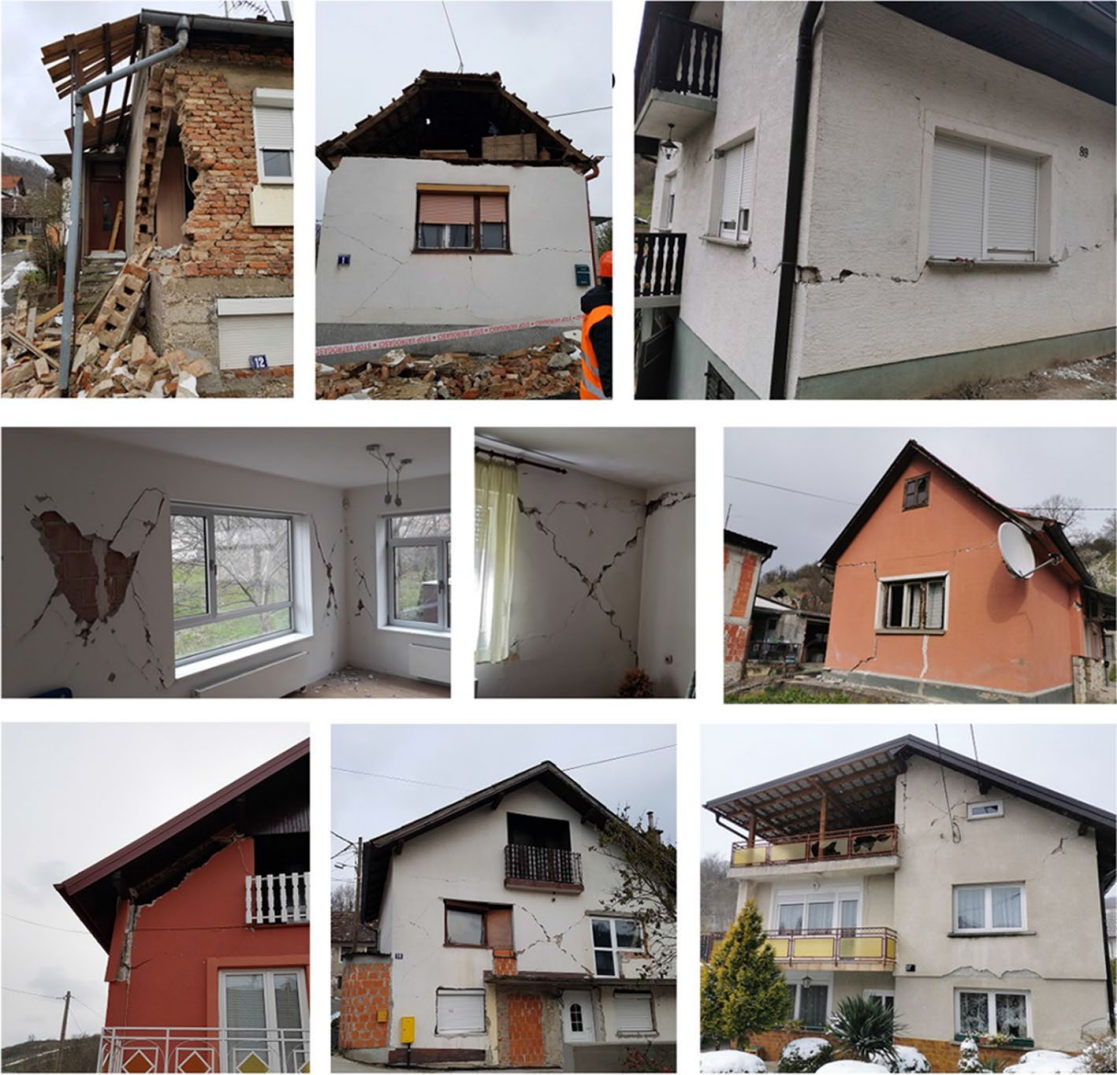
Damage to residential houses in the epicentral region (authors: Luka Božić, Mario Uroš and CCEE 2020)

*Damage to buildings in the epicentral area:* two of the Zagreb neighborhoods are located in the epicentral area, Markuševac and Čučerje. The residential buildings in these areas were built mainly after WW2, most of them with a single residential occupancy. The structural type is mostly brick masonry, reinforced concrete frame with infill brick walls, or a hybrid system without any construction standard. The light roofing material is generally placed or loosely tied at the top edge of the walls. The floor slabs are mainly of prefabricated composite elements resting on the bearing walls, rarely of wooden joists and, in the last decades, of reinforced concrete slabs. The foundation consists of a continuous poured concrete footing and foundation wall or of stone masonry in case of older houses. The brick infill or bearing walls have significant in-plane stiffness, which contributes to the overall stiffness and to reduced deflection to lateral loads. As a result, diagonal cracks were typically observed in all damaged buildings. Damage was most frequent in buildings not properly designed, which have undergone interventions in the structural system (reconstruction, adaptation, enlargement) and/or which were built illegally without the required building permit and safety inspections. Illegal construction is a problem that plagues communities across Croatia. More than 100,000 applications for legalization were recently submitted in the Zagreb region alone. In Fig. 26 are shown several examples of typical damage to those buildings.

## Conclusions

The 2020 M_w_5.4 Zagreb earthquake and the series of aftershocks generated considerable damage to buildings and municipal infrastructure. One person died and 26 people were reported severely injured. The most damage was sustained by masonry buildings with residential occupancy in the historic downtown Zagreb and certain newer improperly designed buildings in the epicentral area. A number of hospitals, churches and other cultural heritage buildings were among the damaged structures. Modern buildings designed with seismic provisions performed well under the earthquake shaking. The earthquake prompted a national scale emergency and thanks to the rapid and important decisions made at state and municipal level, normal conditions were rapidly reestablished. A comprehensive recovery process was instigated expected to extend for years to come.

A snapshot of the pre-earthquake organizational set-up of the public safety framework was fully explained providing the basis for understanding of the emergency response and the ongoing recovery process. The surprisingly heavy social impacts were amplified by a combination of circumstances such as damage concentrated in the very centre of Zagreb, the number of people requiring alternative accommodation, the relatively cold weather and the ongoing Covid-19 pandemic. The typical observed damage to buildings was mainly to non-structural components: damage or collapse of chimneys and attic gable walls, damage to wooden roof structure, detached and falling decorative elements and architectural finishes, cracks in ceilings and partition walls, cracking and partial collapse of lintel and vault sections; and to structural components: in-plane shear cracking and rarely out-of-plane collapse of bearing walls, damage to stairways and to floor slabs due to differential movements of structural elements. About 5,000 of the inspected buildings were with short term repairable damage (temporary unusable), 1300 were with serious structural damage (unusable) with reparable damage or scheduled for demolition, and approximately 20,000 buildings remained usable after the earthquake without or with minor reparations. It is currently estimated that approximately 17,000 buildings were affected in a certain degree by the earthquake. The total economic loss amounts to more than 10B euros, mainly due to reconstruction costs related to the direct physical damage.

Although of moderate magnitude, the Zagreb March 2020 earthquake sent a clear warning to Croatian decision makers that, to guarantee safety and security of citizens and sustainably development of the country, sound and long-term national disaster prevention policies have to be adopted and promoted. They should aim first of all at continuous increasing of the public awareness of own exposure and vulnerability to seismic disasters and at enhancing of all the components of the community resilience, especially those in the preparedness and mitigation phase.

## Data Availability

All data sources are cited in the manuscript and references are provided.
